# Polygenic risk scores for Alzheimer’s disease, and academic achievement, cognitive and behavioural measures in children from the general population

**DOI:** 10.1093/ije/dyz080

**Published:** 2019-05-05

**Authors:** Roxanna Korologou-Linden, Emma L Anderson, Hannah J Jones, George Davey Smith, Laura D Howe, Evie Stergiakouli

**Affiliations:** 1 Medical Research Council Integrative Epidemiology Unit (IEU) at the University of Bristol, Bristol, UK; 2 Population Health Sciences, Bristol Medical School, Bristol, UK; 3 Centre for Academic Mental Health, Population Health Sciences, Bristol Medical School, Bristol, UK; 4 NIHR Biomedical Research Centre at University Hospitals Bristol, Bristol, UK; 5 School of Oral and Dental Sciences, University of Bristol, Bristol, UK

**Keywords:** ALSPAC, Alzheimer’s disease, behavioural, cognitive, polygenic risk scores

## Abstract

**Objective:**

Several studies report a polygenic component of risk for Alzheimer’s disease. Understanding whether this polygenic signal is associated with educational, cognitive and behavioural outcomes in children could provide an earlier window for intervention.

**Methods:**

We examined whether polygenic risk scores (PRS) at varying *P*-value thresholds in children from the Avon Longitudinal Study of Parents and Children were associated with academic achievement, cognitive and behavioural measures in childhood and adolescence.

**Results:**

We did not detect any evidence that the genome-wide significant PRS (5x10^-8^) were associated with these outcomes. PRS at the highest *P*-value threshold examined (*P* ≤ 5x10^-1^) were associated with lower academic achievement in adolescents (Key Stage 3; β: -0.03; 95% confidence interval: -0.05, -0.003) but the effect was attenuated when single nucleotide polymorphisms (SNPs) associated with educational attainment were removed. These PRS were associated with lower IQ (β: -0.04; 95% CI: -0.07, -0.02) at age 8 years with the effect remaining after removing SNPs associated with educational attainment.

**Conclusions:**

SNPs mediating the biological effects of Alzheimer’s disease are unlikely to operate early in life. The evidence of association between PRS for Alzheimer’s disease at liberal thresholds and cognitive measures suggest shared genetic pathways between Alzheimer’s disease, academic achievement and cognition.

**Key Messages**

- This is the first time that the effect of genetic variants for Alzheimer’s disease on academic achievement, cognitive and behavioural measures are being investigated in a large sample of children.
- Genetic variants most strongly associated with Alzheimer’s disease are not associated with any of examined outcomes.
- Alzheimer’s disease may share genetic pathways with cognition and academic achievement, as indicated by findings at liberal *P*-value thresholds.
- Individuals with substantially increased risk of Alzheimer’s disease later in life have similar academic achievement to other individuals in the population.
- Our study suggests that the preclinical effects for Alzheimer’s disease are unlikely to operate in childhood.

## Introduction

Alzheimer’s disease is a heritable neurodegenerative disease which, in addition to other dementia forms, affects 47 million individuals worldwide.^1^ The long prodromal phase is characterized by cognitive decline and behavioural disturbances.^2^^,^^3^ As Alzheimer’s disease exerts a heavy socioeconomic burden,^4^ identifying modifiable factors earlier in life is important for preventing or delaying the onset of the disease.

Genome-wide association studies (GWAS)^5^ have identified several single nucleotide polymorphisms (SNPs) associated with late-onset Alzheimer’s disease, all exerting low to modest effects [except for the ε4 allele in the apolipoprotein E (*ApoE*) gene)].^6^^,^^7^ The effects of common genetic risk variants for complex diseases, including Alzheimer’s disease, can be considered *en masse* to calculate a polygenic risk score (PRS) for disease.^8–10^ These scores can be used as an indicator of genetic risk for Alzheimer’s disease (irrespective of whether an individual will develop Alzheimer’s disease) to investigate the genetic overlap between Alzheimer’s disease and other diseases or traits. The overall SNP heritability of Alzheimer’s disease (24–35%) identified in GWAS^11^ is higher when SNPs of small effect size are also considered, indicating that there are many SNPs below the genome-wide level of significance contributing to increasing genetic risk for Alzheimer’s disease.^8^

Pathophysiological changes resulting in gradual cognitive and functional decline can occur more than two decades before the onset of clinical symptoms.^12^^,^^13^ This presents a challenge in the development of effective treatments and highlights the need for intervention preceding the initiation of the disease process. There is an established association between PRS for Alzheimer’s disease, cognitive outcomes and educational attainment in adults.^14–16^ However, the association between academic achievement, as well as cognitive outcomes, and behavioural difficulties in young ages is understudied.

In our study, we investigated whether a PRS for Alzheimer’s disease is associated with academic achievement at Key Stages 3, 4 and 5, childhood IQ at 8 and 15 years and behavioural difficulties at 9 and 12 years using a large population sample.

## Methods

### Participants

The Avon Longitudinal Study of Parents and Children (ALSPAC) is a prospective birth cohort study which recruited pregnant women residing in the former Avon Health Authority area with expected delivery dates between April 1991 and December 1992; 14 541 pregnant women were initially enrolled, with 14 062 children born. A detailed description of the cohort has been published previously.^17^^,^^18^ Detailed information on health and development of children and their parents was collected from regular clinic visits and completion of questionnaires. The study website contains details of all the data that are available through a fully searchable data dictionary [http://www.bris.ac.uk/alspac/researchers/data-access/data-dictionary/]. Ethical approval was obtained from the ALSPAC Law and Ethics Committee and the local ethics committees.

### ALSPAC genetic data

A total of 9912 ALSPAC children were genotyped on the Illumina HumanHap550-quad SNP genotyping platform. After quality control (QC) assessment published elsewhere,^19^^,^^20^ imputation and restriction to one child per family, genetic data were available for 7977 individuals (QC procedures in Supplementary material, available as Supplementary data at *IJE* online).

### Polygenic risk scores

PRS were computed according to the method described by the International Schizophrenia Consortium^10^ based on the summary statistics from the GWAS of Alzheimer’s disease by the IGAP consortium.^5^ Details about IGAP and PRS are in the Supplementary material, available as Supplementary data at *IJE* online. Our main analysis focused on *P*-value thresholds *P* ≤ 5x10^-8^, 5x10^-2^ and 5x10^-1^, although more thresholds were tested (Supplementary material, available as Supplementary data at *IJE* online). The number of SNPs in the PRS at each *P*-value threshold is provided in Supplementary Table 1, available as Supplementary data at *IJE* online. A list of 19 SNPs used to generate the PRS at the genome-wide significant threshold is provided in Table 1.

**Table 1.** SNPs included in PRS with a genome-wide significance threshold as reported in IGAP stage 1

Marker	Chromosome	Position	Nearest gene	A1	A2	OR
rs6656401	1	207692049	CR1	A	G	1.17
rs6733839	2	127892810	BIN1	T	C	1.21
rs35349669	2	234068476	INPP5D	T	C	1.07
rs190982	5	88223420	MEF2C	G	A	0.92
rs10948363	6	47487762	CD2AP	G	A	1.10
rs2718058	7	37841534	NME8	G	A	0.93
rs1476679	7	100004446	ZCWPW1	C	T	0.92
rs11771145	7	143110762	EPHA1	A	G	0.90
rs28834970	8	27195121	PTK2B	C	T	1.10
rs10838725	11	47557871	CELF1	C	T	1.08
rs983392	11	59923508	MS4A6A	G	A	0.90
rs10792832	11	85867875	PICALM	A	G	0.88
rs11218343	11	121435587	SORL1	C	T	0.76
rs17125944	14	53400629	FERMT2	C	T	1.13
rs10498633	14	92926952	SLC24A4	T	G	0.90
rs4147929	19	1063443	ABCA7	A	G	1.14
rs429358/rs7412	19	45411941/45412079	APOE	ε4	ε2/3	3.86/1.47
rs7274581	20	55018260	CASS4	C	T	0.87

Additional information provided: chromosomal and base pair position, nearest gene, minor allele (A1) and major allele (A2), odds ratio (OR).

### Measures

#### Academic achievement

Academic achievement measures were attained through linkage to compulsory UK educational assessments from the National Pupil Database^21^ and were evaluated at three time points during the pupils’ education; Key Stages 3, 4 and 5. Key Stage 3 national tests are taken when children are 14 years old and include English, Mathematics and Science assessments. For Key Stage 4, General Certificate of Secondary Education (GCSE) examinations determine transition into post-compulsory education. Students study up to 12 subjects (eight on average) of which some are compulsory (e.g. English and Mathematics). The Key Stage 4 scores were analysed as the total point score, which is capped at the student’s eight best GCSE (and equivalent) qualifications.^22^ For Key Stage 5, examinations are taken when the students are 18 years old (A levels) and range from A* to E. Test scores at all Key Stages were provided as a scaled total points score. A binary variable was generated to investigate whether PRS was associated with progression to Key Stage 5 tests for children with available GCSE results.

#### Intelligence quotient

Total IQ scores were collected when children were 8 years old, using the computerized version of the Wechsler Intelligence Scale for Children (WISC-III).^23^ Verbal and performance subtests were administered, their scores were scaled to age, and the total IQ scores derived. At 15 years, children were administered the Vocabulary and Matrix Reasoning subcategories of the Wechsler Abbreviated Scale of Intelligence (WASI).^24^ The verbal, performance and total IQ scores are normative IQs, with a mean of 100 and a standard deviation of 15.^24^ Mean IQ differed from age 8 (IQ = 105.0) to age 15 (IQ = 92.4). A thorough investigation was carried out by ALSPAC, and it was concluded that there were no systematic errors in the way the WASI (or the WISC) was scored. These tests are designed for different age ranges and cannot be interpreted interchangeably or to reflect change over time.^25^

#### Behavioural problems

Behavioural problems in childhood were measured by the Strengths & Difficulties Questionnaire (SDQ),^26^ completed by mothers when the children were 9 and 12 years old. More details on SDQ can be found in Supplementary material, available as Supplementary data at *IJE* online. Due to the skewed nature of the SDQ variables (with most children demonstrating no problems), the five SDQ subscales were dichotomized as in previous studies.^27^^,^^28^

#### Examining the association between the PRS and the outcomes

Linear and ordinary logistic regression models were used for continuous and categorical outcomes, respectively. For the cognitive outcomes (IQ, academic achievement), z-scores were calculated to enable comparison of the magnitude of regression coefficients across outcomes and time points. Models were adjusted for age, sex and the first three ancestry-informative principal components (Model 1). Analyses were performed in Stata 14.^29^ As the outcome measures are highly interdependent, we used principal component analysis (PCA) to extract factors, using a cut-off threshold of an eigenvalue of ≥1 (Kaiser rule).^30^ Our results were interpreted according to the American Statistical Association guidance,^31^^,^^32^ by presenting raw summary statistics and using PCA to assist with interpreting our findings.

#### Sensitivity analysis

Results from Model 1 were compared with those based on scores that excluded SNPs within the *ApoE* gene (Chr. 19: 44 400–46 500 kb) (Model 2) due to the large effect sizes within that region, which may have been driving any observed associations. They were also compared with scores including the two SNPs tagging the *ApoE* gene (rs7412 and rs429358) (Supplementary material, available as Supplementary data at *IJE* online) (Model 3). To exclude the possibility that the effect of the PRS on academic achievement and cognitive outcomes is driven by SNPs associated with educational attainment, we repeated the analysis with a PRS excluding SNPs associated with educational attainment. at *P* ≤ 5x10^-8^ and at *P* ≤ 5x10^-2^. The number of SNPs removed at each PRS *P*-value threshold is provided in Supplementary Table 2, available as Supplementary data at *IJE* online. We also compared indicators of socioeconomic status (SES) for participants with and without genetic data (Supplementary Table 3, available as Supplementary data at *IJE* online).

## Results

### Sample description

The number of children with genetic data and total IQ scores was 5300 at 8 years and 3724 at 15 years. The number of children with educational outcomes was 3990 at 14 years (Key Stage 3) and 6535 at 15 years (Key Stage 4). Among the 6535 children who sat GCSE examinations, 3977 (61%) children also sat Key Stage 5 examinations. IQ scores and academic achievement variables were highly correlated, as described in Supplementary Table 4, available as Supplementary data at *IJE* online. A total of 5525 children had genetic data and a SDQ score. Detailed descriptive statistics are provided in Tables 2 and 3. We found four components to have an eigenvalue above 1 (adjusted_ *P*-value = 1.25x10^-2^). In total, these principal components explained 66% of variation in all the outcomes examined.

**Table 2.** Characteristics of ALSPAC children with available genetic and IQ, or education data

IQ/education measure	*N*	% male	Mean age in years (SD)	Mean score (SD)
IQ at age 8	5300	49.7%	8.6 (0.3)	105.0 (16.4)
IQ at age 15	3724	48.0%	15.4 (0.3)	92.4 (13.1)
Key Stage 3	6029	50.8%	14.1 (0.3)	106.5 (24.3)
Key Stage 4	6535	50.5%	15.0 (0.04)	133.8 (3.8)
Key Stage 5	3990	45.9%	16.3 (0.6)	764.3 (252.7)

ALSPAC, Avon Longitudinal Study of Parents and Children; IQ, intelligence quotient; SD, standard deviation.

**Table 3.** Descriptive statistics for SDQ at age 9

Outcome	Range	Cut-off indicating problems	*N*	*N* (%) with behavioural problems
Total difficulties	0 to 40	≥17	5525	246 (4.45%)
Hyperactivity	0 to 10	≥7	5547	434 (7.82%)
Emotional symptoms	0 to 10	≥5	5532	349 (6.31%)
Conduct problems	0 to 10	≥4	5546	378 (6.82%)
Peer problems	0 to 10	≥4	5541	441 (7.96%)
Prosocial behaviours	0 to 10	≤6	5553	817 (14.7%)

SDQ, strengths and difficulties questionnaire.

### Academic achievement

At a SNP inclusion threshold of *P *≤ 5 x 10^-1^, there was evidence that a PRS was associated with lower total points at Key Stage 3 [β: -0.03; 95% confidence interval (CI): -0.05, -0.003, *P* = 0.03, Table 4] and lower total points at Key Stage 4 (β: -0.03; 95% CI: -0.05, -0.003, *P * = 0.03, Table 4). The direction of effect sizes was similar for SNP inclusion threshold of *P* ≥ 5x10^-2^ (Table 4). The evidence of association when considering only genome-wide significant SNPs (*P *≤ 5x10^-8^) was weak (Table 4). We could not detect any evidence that a PRS at the examined thresholds was associated with increased odds of individuals with GCSE results sitting Key Stage 5 examinations (Supplementary Table 6, available as Supplementary data at *IJE* online).

**Table 4.** Associations between PRS for Alzheimer’s disease and educational, cognitive and behavioural measures

	*P*-value threshold for SNP inclusion								
Outcome	*P* = 5 x 10^-8^ (19 SNPs)			*P* = 5 x 10^-2^ (45 040)			*P* = 5 x 10^-1^ (240 803 SNPs)		
Educational	β (95% CI)	***P***	**R^2^**	β **(95% CI)**	***P***	**R^2^**	β** (95% CI)**	***P***	**R^2^**
Key Stage 3 points	0.01 (-0.01, 0.04)	0.33	2.2 x 10^-3^	−0.002 (-0.03, 0.02)	0.87	2.1 x 10^-3^	−0.03 (-0.05, -0.003)	0.03	2.9 x 10^-3^
Key Stage 4 points	0.001 (-0.02, 0.02)	0.96	3.6 x 10^-3^	−0.01 (-0.04, 0.01)	0.31	3.7 x 10^-3^	−0.03 (-0.05, -0.003)	0.03	4.3 x 10^-3^
Key Stage 5 points	0.01 (-0.02, 0.04)	0.55	1.6 x 10^-3^	−0.01 (-0.04, 0.02)	0.46	1.6 x 10^-3^	−0.03 (-0.06, 0.003)	0.08	2.2 x 10^-3^
Cognitive									
Total IQ, age 8	0.0002 (-0.03, 0.03)	0.99	2.7 x 10^-4^	−0.03 (-0.06, -0.01)	0.01	1.5 x 10^-3^	−0.04 (-0.07, -0.02)	0.002	2.1 x 10^-3^
Total IQ, age 15	−0.01 (-0.04, 0.02)	0.44	1.3 x 10^-3^	−0.01 (-0.05, 0.02)	0.38	1.3 x 10^-3^	−0.02 (-0.05, 0.01)	0.20	1.5 x 10^-3^
	**OR (95% CI)**	***P***	**R^2^**	**OR (95% CI)**	***P***	**Pseudo-R^2^**	**OR (95% CI)**	***P***	**Pseudo-R^2^**
Behavioural									
Total difficulties, age 9	1.00 (0.89, 1.14)	0.95	5.3 x 10^-4^	1.02 (0.90, 1.16)	0.78	5.6 x 10^-4^	1.05 (0.92, 1.19)	0.46	7.9 x 10^-4^
Prosocial, age 9	0.97 (0.90, 1.04)	0.36	1.4 x 10^-3^	0.98 (0.91, 1.06)	0.58	1.3 x 10^-3^	1.00 (0.93, 1.07)	0.95	1.2 x 10^-3^
Hyperactivity, age 9	0.99 (0.90, 1.09)	0.86	1.3 x 10^-3^	1.02 (0.92, 1.12)	0.73	1.3 x 10^-3^	1.05 (0.96. 1.16)	0.29	1.6 x 10^-3^
Emotional symptoms, age 9	0.94 (0.84, 1.05)	0.25	2.9 x 10^-3^	1.00 (0.90, 1.11)	0.96	2.3 x 10^-3^	1.07 (0.96, 1.19)	0.23	2.9 x 10^-3^
Conduct problems, age 9	0.96 (0.86, 1.07)	0.43	9.5 x 10^-4^	0.99 (0.89, 1.10)	0.88	7.3 x 10^-4^	1.01 (0.91, 1.13)	0.79	7.5 x 10^-4^
Peer problems, age 9	1.01 (0.91, 1.11)	0.91	4.5 x 10^-4^	0.93 (0.85, 1.03)	0.16	1.1 x 10^-3^	0.97 (0.88, 1.07)	0.53	5.8 x 10^-4^
Total difficulties, age 12	1.01 (0.90, 1.14)	0.85	1.7 x 10^-3^	1.05 (0.93, 1.17)	0.46	2.0 x 10^-3^	1.04 (0.92, 1.16)	0.56	1.9 x 10^-3^
Prosocial, age 12	0.93 (0.86, 1.00)	0.05	2.0 x 10^-3^	1.04 (0.97, 1.13)	0.28	1.4 x 10^-3^	1.10 (1.02, 1.18)	0.02	2.4 x 10^-3^
Hyperactivity, age 12	0.97 (0.87, 1.08)	0.56	4.9 x 10^-4^	1.06 (0.95, 1.18)	0.30	7.7 x 10^-4^	1.07 (0.96, 1.20)	0.20	1.0 x 10^-3^
Emotional symptoms, age 12	1.07 (0.96, 1.20)	0.19	1.4 x 10^-3^	1.12 (1.00, 1.25)	0.05	2.3 x 10^-3^	1.16 (1.04, 1.26)	0.01	3.5 x 10^-3^
Conduct problems, age 12	0.99 (0.89, 1.10)	0.81	1.6 x 10^-3^	1.04 (0.94, 1.16)	0.46	1.8 x 10^-3^	1.05 (0.94, 1.16)	0.42	1.8 x 10^-3^
Peer problems, age 12	0.96 (0.87, 1.06)	0.43	3.0 x 10^-4^	0.96 (0.87, 1.06)	0.40	3.3 x 10^-4^	1.00 (0.91, 1.11)	0.92	9.4 x 10^-5^

PRS, polygenic risk scores; SNPs, single nucleotide polymorphisms.

Adjusted *P*-value threshold = 0.0125

#### Cognitive measures

At 8 years and using an SNP inclusion threshold of *P*≤5x10^-1^, there was strong evidence of a PRS being associated with lower total IQ (β: -0.04; 95% CI: -0.07, -0.02, *P * = 0.002, Table 4) as well as lower verbal (β: -0.04; 95% CI: -0.07, -0.01, *P * = 0.003) and performance IQ (β: -0.03; 95% CI: -0.06, -0.01, *P * = 0.01, Supplementary Table 7, available as Supplementary data at *IJE* online). The pattern of results was similar for a threshold of *P*≥5x10^-2^, whereas the evidence of association when considering only genome-wide significant SNPs (*P*≤5x10^-8^) was weak (Table 4). At 15 years, we could not detect any evidence of association between PRS for Alzheimer’s and total IQ or IQ domains, using either a genome-wide significant SNP threshold or more liberal SNP inclusion thresholds (Table 4; Supplementary Table 8, available as Supplementary data at *IJE* online). However, the direction of effects was the same as for age 8, with PRS suggesting lower total IQ (β: -0.02; 95% CI: -0.05, 0.01, *P * = 0.20 at *P*≤5x10^-1^, Table 4). Also, there was some evidence of association between PRS at SNP inclusion thresholds of *P*≥5x10^-2^ with matrix reasoning scores (Supplementary Table 8, available as Supplementary data at *IJE* online).

#### Behavioural difficulties

At all of the examined *P*-value thresholds, there was weak evidence that a PRS was associated with the SDQ components at 9 years (Supplementary Table 9, available as Supplementary data at *IJE* online). At 12 years and an SNP inclusion threshold *P *≤5x10^-1^, there was some evidence that a PRS may be associated with the prosocial and emotional score (Table 4). At an SNP inclusion threshold of *P*≤5x10^-2^, there was evidence of association for a PRS with higher odds of having an abnormal emotional symptoms score (OR: 1.16; 95% CI: 1.04, 1.26, *P * = 0.01 at *P*≤5x10^-1^, Table 4). We could not detect any evidence for association between a PRS at the genome-wide significant threshold and the SDQ domains.

#### Sensitivity analysis

The size and direction of effect estimates for PRS excluding the SNPs within the *ApoE* gene were similar for most outcomes (Supplementary Tables 5–10, available as Supplementary data at *IJE* online). The direction of effects and variance explained by the two SNPs tagging the *ApoE* region was similar to that explained by the genome-wide significant PRS (with *ApoE*) for most outcomes (Supplementary Tables 5–10, available as Supplementary data at *IJE* online). Results for PRS excluding the SNPs associated with educational attainment at 5x10^-8^ were similar to results in the main analysis. When SNPs associated with educational attainment at *P*≤5x10^-2^ were removed, evidence of association was attenuated (Supplementary Tables 11–18, available as Supplementary data at *IJE* online). In our comparison of participants with and without genetic data in terms of SES, availability of genetic data was associated with indicators of SES (Supplementary Table 3, available as Supplementary data at *IJE* online).

## Discussion

This is one of the first studies investigating whether common genetic variants predisposing to a higher risk of Alzheimer’s disease are associated with educational, cognitive and behavioural measures in children from a general population cohort study. Our findings indicate little evidence for association of the PRS at the genome-wide significant threshold with cognitive measures, academic achievement and behavioural difficulties. We found that PRS at liberal *P*-value thresholds were associated with lower academic achievement at 14 and 15 years, as well as lower total IQ and IQ domain scores at 8 years.

**Figure 1.**
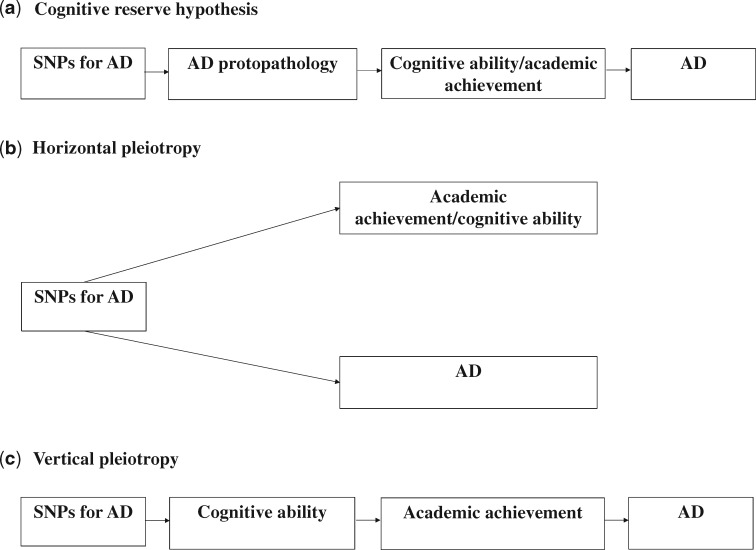
Potential mechanisms of associations between PRS for Alzheimer’s disease and cognitive ability/academic achievement. Please note that these are not intended to be directed acyclic graphs. AD denotes Alzheimer’s disease. In panel (a), genetic variants for Alzheimer’s disease cause Alzheimer’s disease protopathology, which manifests as lower IQ and poorer academic achievement at young ages. This could result in reduced ability to tolerate and compensate for Alzheimer’s disease pathology. Panel (b) describes the situation where genetic variants that increase predisposition for Alzheimer’s disease affect academic achievement and/or cognitive ability through an independent pathway (horizontal pleiotropy). In panel (c), genetic variants used to instrument Alzheimer’s disease have their primary effect on Alzheimer’s disease through academic achievement and/or cognitive ability rather than vice versa.

The use of PRS with *P*-value thresholds of varying strengths, as well as accounting for genetic variants associated with educational attainment, allowed for the testing of three possible mechanisms by which genetic variants for Alzheimer’s disease may affect the examined outcomes. The first possibility is that risk variants for Alzheimer’s are associated with disease protopathology in early life which manifests as lower IQ and poorer academic achievement in early life. Lower IQ and poorer academic achievement at young ages may reduce brain and cognitive reserve, which could result in reduced ability to tolerate and compensate for Alzheimer’s disease pathology through structural differences in the brain and/or pre-existing cognitive processing approaches/activation of compensatory mechanisms, respectively known as the brain/cognitive reserve hypothesis^33^ (Figure 1a). This is not supported by our findings, since genetic variants most strongly associated with Alzheimer’s disease (i.e. 5 x 10^-8^) should also have been associated with cognitive and educational measures in childhood to support this hypothesis. The second possibility is the presence of horizontal pleiotropy.^34^^,^^35^ SNPs associated with Alzheimer’s disease could be associated with lower academic achievement and lower cognitive ability through biological pathways that are completely unrelated to the disease (Figure 1b). This could be the case at least for cognitive ability, since evidence of association between the PRS at liberal thresholds and cognitive ability remained after the removal of SNPs associated with educational attainment.

Furthermore, a recent Mendelian randomization study examining the relationship between intelligence and Alzheimer’s disease identified some evidence of horizontal pleiotropy.^36^ For education, this explanation is not supported by our findings or by Mendelian randomization studies,^36–38^ which indicated a causal effect of education on Alzheimer’s disease without detecting the presence of pleiotropy.^36^^,^^38^ The third possibility is the presence of vertical pleiotropy^34^^,^^35^; genetic variants associated with Alzheimer’s disease are only associated with the disease because they reduce educational attainment and/or cognitive ability (Figure 1c). Our findings support this possibility, as the observed association between PRS for Alzheimer’s disease and academic achievement is fully attenuated when the genetic variants associated with educational attainment are removed.

We did not detect consistent associations between the PRS at liberal *P*-value thresholds across all time points tested. This could be due to either: (i) lower participation and consequently lower power to detect associations due to differences in sample size across ages; (ii) varying impact of environmental factors on behavioural changes across development; or (iii) difference in measures.

### Comparison with other studies

In agreement with a previous study showing no effect of *ApoE* on cognitive outcomes in ALSPAC children, we did not find any evidence that the genome-wide significant PRS was associated with cognition.^39^ A recent study^40^ in children and adolescents from two independent cohorts in Brazil (*N* = 716) found that a PRS for Alzheimer’s disease was associated with lower scores in non-declarative memory exercises at *P* ≤ 0.01 and with reading and writing at more liberal *P*-value thresholds. They did not find strong evidence for cognitive tasks or brain structure in a comparable sample of adolescents (*N* = 1029, mean age 15 years). Our findings are also in agreement with those from adult populations for which there is an established association between a PRS for Alzheimer’s disease and adverse cognitive outcomes.^15^^,^^16^ In adults from the UK Biobank, there is an association between a higher PRS for Alzheimer’s disease with lower cognitive performance (*P*≥1x10^-2^) and educational attainment (*P*≥5x10^-2^).^16^ Furthermore, a phenome-wide analysis^41^ using linkage disequilibrium score regression showed negative genetic correlations between Alzheimer’s disease and cognitive outcomes. In line with our findings, this study did not detect associations between a PRS for Alzheimer’s disease at *P* ≤ 0.30 and different SDQ components.

### Strengths and limitations

Our study benefits from using a large discovery GWAS of individuals with a clinical diagnosis of late-onset Alzheimer’s and a large sample of children representative of the population as a target sample. Our study differs from many earlier studies, in that we used academic achievement on a continuous scale, rather than educational attainment which is traditionally measured on a categorical scale. Thus, our results are far more precise and potentially very well powered to pick up effects. The use of the PRS in children allowed us to perform this investigation without the selection bias present in late life studies.

The inherent limitation of using PRS is the limited amount of phenotypic variance they explain, which is also true for this study (<1%). Genome-wide complex trait analysis has shown that variants achieving genome-wide significance for Alzheimer’s currently explain only 16.3% of phenotypic variance. When all SNPs in the dataset are included, the amount of phenotypic variance explained increases to 53.2%.^42^ We used a more liberal threshold for SNP inclusion than that of genome-wide significance because, although it might introduce noise,^43^ it increases power to detect individuals at highest/lowest risk.^9^ Another limitation of this study could be the differential attrition or non-participation by PRS, which has been shown to introduce collider bias into studies for psychiatric disorders. A recent study in addressing the issue of selection bias in genetic studies did not detect evidence for an association between PRS for Alzheimer’s disease and non-participation in ALSPAC.^44^

Our study is the first to examine the association between genetic variants for Alzheimer’s disease and both educational, cognitive and behavioural outcomes in childhood and adolescence in such a large sample. We show with great precision that individuals with substantially increased risk of Alzheimer’s disease later in life have similar educational attainment to other individuals in the population. This suggests that the risk factors for Alzheimer’s disease play a role after adolescence.

## Funding

This work was supported by a grant from the BRACE Alzheimer’s charity (BR16/028). The UK Medical Research Council and Wellcome (Grant ref: 102215/2/13/2) and the University of Bristol provide core support for ALSPAC. The collection of the WASI variable was supported by a grant from the Wellcome Trust (Grant ref: 076467/Z/05/Z). GWAS data were generated by Sample Logistics and Genotyping Facilities at Wellcome Sanger Institute and LabCorp (Laboratory Corporation of America) using support from 23andMe. L.D.H. and E.L.A. are supported by fellowships from the UK Medical Research Council (MR/M020894/1 and MR/P014437/1, respectively). All authors work in a unit that receives funding from the University of Bristol and the UK Medical Research Council (MC_UU_00011-1). This publication is the work of the authors, and E,S, will serve as a guarantor for the contents of this paper. 

## Supplementary Material

dyz080_Supplementary_Material
