# Supplementary material for: Polygenic risk scores for Alzheimer’s disease, and academic achievement, cognitive and behavioural measures in children from the general population
Source: Int J Epidemiol. 2019 May 5;48(6):1972–80. doi: 10.1093/ije/dyz080 (PMC6929531; doi:10.1093/ije/dyz080)
Supplement: dyz080_Supplementary_Material [file dyz080_supplementary_material.docx]

# **Supplementary Material**

# **Methods**

## **Genetic data**

A total of 9912 ALSPAC children [1,2] were genotyped on the Illumina HumanHap550-quad SNP genotyping platform. The resulting raw genome-wide data were subjected to standard quality control methods. Exclusion criteria of individuals from further analysis was based on gender mismatches, minimal or excessive heterozygosity, disproportionate levels of individual missingness (>3%) and evidence of cryptic relatedness (>10% of shared alleles identical by descent). Furthermore, all individuals with non-European ancestry were removed based on assessment by multidimensional scaling analysis including HapMap 2 individuals. SNPs with a minor allele frequency of <1%, Impute2 information quality metric of <0.8, a call rate of <95% or evidence of violating the Hardy-Weinberg equilibrium (p< 5x10^-7^) were removed. Related subjects that passed all other quality control thresholds were retained during subsequent phasing and imputation. Following quality control procedures and imputation and restricting to 1 child per family, genetic data was available for 7,977 children.

**Discovery sample**

The International Genomics of Alzheimer's Project (IGAP) is a large two-stage study based upon genome-wide association studies (GWAS) on individuals of European ancestry. In stage 1, IGAP used genotyped and imputed data on 7,055,881 single nucleotide polymorphisms (SNPs) to meta-analyse four previously-published GWAS datasets consisting of 17,008 Alzheimer's disease cases and 37,154 controls performed by GERAD (Genetic and Environmental Risk in Alzheimer’s disease), EADI (The European Alzheimer's disease Initiative), CHARGE (Cohorts for Heart and Aging Research in Genomic Epidemiology), and ADGC (Alzheimer Disease Genetics Consortium) [3]. Complete details of each study, as well as the samples and methodologies are reported elsewhere [3–7]. Each dataset was imputed with either Impute 2 [8] or MACH software [9], utilising the 1000 genomes data as a reference panel.

**PRS**

SNPs were removed from the analysis if there was an allelic mismatch between samples (the alleles reported by the IGAP study did not match the alleles present in the ALSPAC sample). Using the ALSPAC study data, pruning was performed (--clump command in PLINK 1.9 genetic analysis tool [10]) using an r^2^ parameter of 0.25 and a physical distance threshold for clumping SNPs of 500 kB. A PRS was calculated for each participant with genetic data. Each score was calculated from the effect size (logarithm (log)odds))-weighted sum of associated alleles within each participant. As the composition of a PRS is a balance between true and null effects [11], scores were constructed using a range of p-value thresholds (p≤5x10^-8^ (genome-wide significant SNPs), 1x10^-3^, 5x10^-2^, 1x10^-1^, and 5x10^-1^). SNPs at the CLU (rs9331896) and the HLA region (rs9271192) were not present in ALSPAC and were omitted from the PRS. The PRS was standardised by subtracting the mean and dividing by the standard deviation.

**SDQ**

The SDQ consists of 25 questions, divided into 5-item sub-categories: hyperactivity, conduct problems, emotional symptoms, peer problems and prosocial behaviour each scored from 0 to 10. A total difficulties score (0 to 40) was computed for the following four subcategories: emotional symptoms, conduct problems, hyperactivity symptoms, and peer problems (excluding prosocial behaviour, for which a higher score is suggestive of fewer behavioural problems) (<http://www.sdqinfo.com>). Abnormal scores for each of the SDQ subcategories were as follows; SDQ total difficulties (score≥17), emotional symptoms (score≥5), conduct problems (score≥4), hyperactivity symptoms (score ≥7), peer problems (score≥4), and prosocial behaviour (score≤6).

| **Table 1.** Number of single nucleotide polymorphism in polygenic risk scores at each p-value threshold | | |
| --- | --- | --- |
| **P-value threshold** | **PRS excluding ApoE** | **PRS including ApoE** |
| 5x10^-8^ | 17 | 19 |
| 1x10^-3^ | 1,890 | 1,965 |
| 5x10^-2^ | 44,893 | 45,040 |
| 1x10^-1^ | 77,162 | 77,329 |
| 5x10^-1^ | 240,560 | 240,803 |

Abbreviations: *ApoE,* apoliprotein E; PRS, polygenic risk scores.

| **Table 2.** Number of single nucleotide polymorphisms (associated with educational attainment at 5x10^-8^ and 5x10^-2^) removed from the PRS for Alzheimer’s disease | | | | |
| --- | --- | --- | --- | --- |
|  | **PRS (with *ApoE*)** | | **PRS (without *ApoE*)** | |
| **PRS p-value threshold** | **No. of SNPs associated with EA at 5x10^-8^** | **No. of SNPs associated with EA at 5x10^-2^** | **No. of SNPs associated with EA at 5x10^-8^** | **No. of SNPs associated with EA at 5x10^-2^** |
| 5x10^-8^ | 0 | 3 | 0 | 2 |
| 1x10^-3^ | 2 | 271 | 2 | 261 |
| 5x10^-2^ | 87 | 5,908 | 87 | 5,894 |
| 1x10^-1^ | 129 | 9,859 | 129 | 9,842 |
| 5x10^-1^ | 288 | 28,846 | 288 | 28,817 |

Abbreviations: EA, educational attainment; no, number; *ApoE, apolipoprotein E ;*PRS, polygenic risk scores.

| **Table 3.** Distribution of the study sample according to availability of children’s genetic data, ALSPAC | | | | | | |
| --- | --- | --- | --- | --- | --- | --- |
|  | **Availability of Child’s Genetic Data** | | | |  | |
|  | **Available** | | **Not available** | | |  |
| **Characteristic** | **No.** | **%** | **No.** | **%** | | **P Value for comparison** |
| **Sex of child** |  |  |  |  | | 0.995 |
| Male | 4,028 | 51.32 | 3,508 | 51.32 | |  |
| Female | 3,821 | 48.68 | 3,327 | 48.68 | |  |
| **Household social class** |  |  |  |  | |  |
| Professional | 960 | 16.71 | 454 | 12.01 | | <0.0001 |
| Managerial & Technical | 2,671 | 46.48 | 1,611 | 42.61 | |  |
| Non-manual | 1,507 | 26.23 | 1,114 | 29.46 | |  |
| Manual | 458 | 7.97 | 451 | 11.93 | |  |
| Part Skilled & Unskilled | 150 | 2.61 | 151 | 3.99 | |  |
| **Maternal education** |  |  |  |  | |  |
| Less than O level | 1,731 | 24.46 | 1,997 | 37.45 | | <0.0001 |
| O level | 2,460 | 34.76 | 1,834 | 34.39 | |  |
| A level | 1,772 | 25.04 | 1,019 | 19.11 | |  |
| Degree or above | 1,115 | 15.75 | 483 | 9.06 | |  |
| **Partners highest educational qualification** |  |  |  |  | |  |
| Less than O level | 2,006 | 29.20 | 2,116 | 41.80 | | <0.0001 |
| O level | 1,505 | 21.90 | 1,035 | 20.45 | |  |
| A level | 1,882 | 27.39 | 1,222 | 24.14 | |  |
| Degree or Above | 1,478 | 21.51 | 689 | 13.61 | |  |
| **Maternal smoking during pregnancy** |  |  |  |  | |  |
| Yes | 1,561 | 21.43 | 1,732 | 29.52 | | <0.0001 |
| No | 5,724 | 78.57 | 4,135 | 70.48 | |  |
|  | ***Total (N)*** | ***Mean (SD)*** | ***Total*** | ***Mean (SD)*** | | **P value** |
| Child gestational age at birth | 7,508 | 39.49 (1.80) | 6,453 | 39.28 (2.08) | | <0.00001 |

| **Table 4** Correlation matrix for examined outcomes | | | | | | |
| --- | --- | --- | --- | --- | --- | --- |
|  | IQ, age 8yrs | IQ, age 15yrs | KS2 points | KS3 points | KS4 points | KS5 points |
| IQ, age 8yrs | 1.00 | 0.60 | 0.36 | 0.60 | 0.59 | 0.38 |
| IQ, age 15yrs | 0.60 | 1.00 | 0.46 | 0.55 | 0.56 | 0.41 |
| KS2 points | 0.36 | 0.46 | 1.00 | 0.62 | 0.61 | 0.32 |
| KS3 points | 0.60 | 0.55 | 0.51 | 1.00 | 0.74 | 0.46 |
| KS4 points | 0.59 | 0.56 | 0.56 | 0.74 | 1.00 | 0.65 |
| KS5 points | 0.38 | 0.41 | 0.32 | 0.46 | 0.65 | 1.00 |

|  |  | **Model 1** | | | **Model 2** | | | **Model 3** | | |
| --- | --- | --- | --- | --- | --- | --- | --- | --- | --- | --- |
| **Outcome** | **Threshold** | **β-coefficient**  **(95% CI)** | **R^2^** | **p** | **β-coefficient**  **(95% CI)** | **R^2^** | **p** | **β-coefficient**  **(95% CI)** | **R^2^** | **p** |
| Key Stage 3 | 5x10^-8^ | 0.01 (-0.01, 0.04) | 2.2x10^-3^ | 0.33 | 0.01 (-0.02, 0.03) | 2.1x10^-3^ | 0.61 | 0.01 (-0.01, 0.04) | 2.2x10^-3^ | 0.42 |
|  | 1x10^-3^ | 0.01 (-0.02, 0.03) | 2.2x10^-3^ | 0.51 | 0.01 (-0.02, 0.04) | 2.2x10^-3^ | 0.43 |  |  |  |
|  | 5x10^-2^ | -0.002 (-0.03, 0.02) | 2.1x10^-3^ | 0.87 | -0.02 (-0.05, 0.01) | 2.5x10^-3^ | 0.12 |  |  |  |
|  | 1x10^-1^ | -0.01 (-0.04, 0.01) | 2.3x10^-3^ | 0.32 | -0.02 (-0.05, 0.002) | 2.6x10^-3^ | 0.07 |  |  |  |
|  | 5x10^-1^ | -0.03 (-0.05, -0.003) | 2.9x10^-3^ | 0.03 | -0.03 (-0.06, -0.01) | 3.2x10^-3^ | 0.01 |  |  |  |
| Key Stage 4 | 5x10^-8^ | 0.001 (-0.02, 0.02) | 3.6x10^-3^ | 0.96 | 0.001 (-0.02, 0.02) | 3.6x10^-3^ | 0.95 | 0.003 (-0.02, 0.03) | 3.6x10^-3^ | 0.83 |
|  | 1x10^-3^ | 0.01 (-0.01, 0.03) | 3.7x10^-3^ | 0.46 | 0.01 (-0.02, 0.04) | 3.6x10^-3^ | 0.54 |  |  |  |
|  | 5x10^-2^ | -0.01 (-0.04, 0.01) | 3.7x10^-3^ | 0.31 | -0.03 (-0.05, -0.001) | 4.2x10^-3^ | 0.04 |  |  |  |
|  | 1x10^-1^ | -0.02 (-0.04, 0.003) | 4.0x10^-3^ | 0.09 | -0.03 (-0.05, -0.002) | 4.2x10^-3^ | 0.03 |  |  |  |
|  | 5x10^-1^ | -0.03 (-0.05, -0.003) | 4.3x10^-3^ | 0.03 | -0.04 (-0.06, -0.01) | 4.9x10^-3^ | 0.003 |  |  |  |
| Key Stage 5 | 5x10^-8^ | 0.01 (-0.02, 0.04) | 1.6x10^-3^ | 0.55 | -0.0001 (-0.03, 0.03) | 1.5x10^-3^ | 0.99 | 0.01 (-0.02, 0.04) | 1.6x10^-3^ |  |
|  | 1x10^-3^ | 0.01 (-0.02, 0.04) | 1.6x10^-3^ | 0.56 | 0.01 (-0.02, 0.04) | 1.6x10^-3^ | 0.46 |  |  |  |
|  | 5x10^-2^ | -0.01 (-0.04, 0.02) | 1.6x10^-3^ | 0.46 | -0.03 (-0.06, 0.003) | 2.3x10^-3^ | 0.07 |  |  | 0.65 |
|  | 1x10^-1^ | -0.03 (-0.06, 0.005) | 2.1x10^-3^ | 0.10 | -0.04 (-0.07, -0.01) | 3.0x10^-3^ | 0.01 |  |  |  |
|  | 5x10^-1^ | -0.03 (-0.06, 0.003) | 2.2x10^-3^ | 0.08 | -0.04 (-0.07, -0.01) | 2.9x10^-3^ | 0.01 |  |  |  |
| CI, confidence interval; PRS, polygenic risk score.  Model 1 is based on the PRS including all independent single nucleotide polymorphisms.  Model 2 is based on the PRS including independent single nucleotide polymorphisms, except for the Apolipoprotein (ApoE) region (Chr. 19; 44,400–46,500 kb).  Model 3 is based on the PRS of the two SNPs tagging the Apolipoprotein (ApoE) region (rs7412 and rs429358).  Adjusted p-value threshold=0.0125. | | | | | | | | | |  |

**Table 5** Association of PRS for Alzheimer’s disease with total points for Key Stages 3-5

| **Table 6** Association of PRS for Alzheimer’s disease and odds of sitting Key Stage 5 exams | | | | | | | | | |
| --- | --- | --- | --- | --- | --- | --- | --- | --- | --- |
|  | **Model 1** | | | **Model 2** | | | **Model 3** | | |
| **Threshold** | **OR (95% CI)** | **Pseudo-R^2^** | **p** | **OR (95% CI)** | **Pseudo-R^2^** | **p** | **OR (95% CI)** | **Pseudo-R^2^** | **p** |
| 5x10^-8^ | 0.95 (0.85, 1.05) | 1.0x10^-3^ | 0.30 | 1.02 (0.92, 1.14) | 6.7x10^-4^ | 0.67 | 0.93 (0.84,1.03) | 1.4x10^-3^ | 0.16 |
| 1x10^-3^ | 0.97 (0.87, 1.07) | 7.6x10^-4^ | 0.52 | 0.94 (0.84, 1.04) | 1.2x10^-3^ | 0.22 |  |  |  |
| 5x10^-2^ | 0.98 (0.88, 1.09) | 6.5x10^-4^ | 0.73 | 0.95 (0.86, 1.06) | 9.1x10^-4^ | 0.38 |  |  |  |
| 1x10^-1^ | 0.96 (0.86, 1.07) | 8.5x10^-4^ | 0.43 | 0.96 (0.87, 1.07) | 7.8x10^-4^ | 0.51 |  |  |  |
| 5x10^-1^ | 0.94 (0.85, 1.05) | 1.1x10^-3^ | 0.26 | 0.98 (0.88, 1.09) | 6.6x10^-4^ | 0.70 |  |  |  |
| CI, confidence interval; OR, odds ratio; PRS, polygenic risk score.  Model 1 is based on the PRS including all independent single nucleotide polymorphisms.  Model 2 is based on the PRS including independent single nucleotide polymorphisms, except for the Apolipoprotein (ApoE) region (Chr. 19; 44,400–46,500 kb).  Model 3 is based on the PRS of the two SNPs tagging the Apolipoprotein (ApoE) region (rs7412 and rs429358).  Adjusted p-value threshold=0.0125. | | | | | | | | | |

| **Table 7** Association of PRS for Alzheimer’s disease with cognitive measures at age 8 | | | | | | | | | |  |
| --- | --- | --- | --- | --- | --- | --- | --- | --- | --- | --- |
|  |  | **Model 1** | |  | **Model 2** | |  | **Model 3** | |  |
| **Outcome** | **Threshold** | **β-coefficient**  **(95% CI)** | **R^2^** | **p** | **β-coefficient**  **(95% CI)** | **R^2^** | **p** | **β-coefficient**  **(95% CI)** | **R^2^** | **p** |
| Total IQ | 5x10^-8^ | -0.001 (-0.03,0.03) | 2.7x10^-4^ | 0.99 | 0.003 (-0.02, 0.03) | 2.8x10^-4^ | 0.82 | -0.001 (-0.03, 0.03) | 2.7x10^-4^ |  |
|  | 1x10^-3^ | -0.01 (-0.03, 0.02) | 3.4x10^-4^ | 0.53 | 0.01 (-0.02, 0.03) | 3.0x10^-4^ | 0.69 |  |  |  |
|  | 5x10^-2^ | -0.03 (-0.06, -0.01) | 1.5x10^-3^ | 0.01 | -0.02 (-0.05, 0.002) | 8.8x10^-4^ | 0.07 |  |  | 0.96 |
|  | 1x10^-1^ | -0.04 (-0.07, -0.02) | 2.1x10^-3^ | 0.002 | -0.04 (-0.07, -0.01) | 2.0x10^-3^ | 0.003 |  |  |  |
|  | 5x10^-1^ | -0.04 (-0.07, -0.02) | 2.1x10^-3^ | 0.002 | -0.04 (-0.07, -0.02) | 2.2x10^-3^ | 0.001 |  |  |  |
| Verbal IQ | 5x10^-8^ | 0.01 (-0.02, 0.03) | 6.2x10^-4^ | 0.64 | 0.02 (-0.01, 0.05) | 9.4x10^-4^ | 0.16 | -0.002 (-0.03, 0.02) | 5.8x10^-4^ |  |
|  | 1x10^-3^ | 0.004 (-0.02, 0.03) | 6.0x10^-4^ | 0.75 | 0.02 (-0.01, 0.05) | 9.2x10^-4^ | 0.17 |  |  |  |
|  | 5x10^-2^ | -0.03 (-0.05, 0.001) | 1.2 x10^-3^ | 0.06 | -0.02 (-0.04, 0.01) | 8.9x10^-4^ | 0.20 |  |  | 0.87 |
|  | 1x10^-1^ | -0.04 (-0.06, -0.01) | 1.9x10^-3^ | 0.01 | -0.03 (-0.06, -0.004) | 1.5x10^-3^ | 0.02 |  |  |  |
|  | 5x10^-1^ | -0.04 (-0.07, -0.01) | 2.2x10^-3^ | 0.003 | -0.03 (-0.06, -0.01) | 1.7x10^-3^ | 0.01 |  |  |  |
| Performance IQ | 5x10^-8^ | -0.004 (-0.03, 0.02) | 2.4x10^-4^ | 0.74 | -0.01 (-0.04, 0.01) | 4.4x10^-4^ | 0.28 | 0.003 (-0.02, 0.03) | 2.3x10^-4^ |  |
|  | 1x10^-3^ | -0.02 (-0.04, 0.01) | 5.5x10^-4^ | 0.18 | -0.01 (-0.04, 0.02) | 3.0x10^-4^ | 0.52 |  |  |  |
|  | 5x10^-2^ | -0.04 (-0.06, -0.01) | 1.5x10^-3^ | 0.01 | -0.03 (-0.05, 0.001) | 9.0x10^-4^ | 0.06 |  |  | 0.80 |
|  | 1x10^-1^ | -0.04 (-0.07, -0.01) | 1.8x10^-3^ | 0.003 | -0.04 (-0.07, -0.02) | 2.0x10^-3^ | 0.002 |  |  |  |
|  | 5x10^-1^ | -0.03 (-0.06, -0.01) | 1.3x10^-3^ | 0.01 | -0.04 (-0.07, -0.02) | 2.1x10^-3^ | 0.002 |  |  |  |
| CI, confidence interval; IQ, intelligence quotient; PRS, polygenic risk score  Model 1 is based on the PRS including all independent single nucleotide polymorphisms.  Model 2 is based on the PRS including independent single nucleotide polymorphisms, except for the Apolipoprotein (ApoE) region (Chr. 19; 44,400–46,500 kb).  Model 3 is based on the PRS of the two SNPs tagging the Apolipoprotein (ApoE) region (rs7412 and rs429358).  Adjusted p-value threshold=0.0125. | | | | | | | | | |  |

| **Table 8** Association of PRS for Alzheimer’s disease with cognitive measures at age 15 | | | | | | | | | |  |
| --- | --- | --- | --- | --- | --- | --- | --- | --- | --- | --- |
|  |  | **Model 1** | | | **Model 2** | | | **Model 3** | | |
| **Outcome** | **Threshold** | **β-coefficient**  **(95% CI)** | **R^2^** | **p** | **β-coefficient**  **(95% CI)** | **R^2^** | **p** | **β-coefficient**  **(95% CI)** | **R^2^** | **p** |
| Total IQ | 5x10^-8^ | -0.01 (-0.04, 0.02) | 1.3x10^-3^ | 0.44 | 0.01 (-0.03, 0.04) | 1.1x10^-3^ | 0.68 | -0.01 (-0.05,0.02) | 1.3x10^-3^ | 0.41 |
|  | 1x10^-3^ | -0.01 (-0.04, 0.02) | 1.3x10^-3^ | 0.43 | 0.02 (-0.02, 0.05) | 1.4x10^-3^ | 0.33 |  |  |  |
|  | 5x10^-2^ | -0.01 (-0.05, 0.02) | 1.3x10^-3^ | 0.38 | -0.003 (-0.03, 0.03) | 1.1x10^-3^ | 0.86 |  |  |  |
|  | 1x10^-1^ | -0.03 (-0.06, 0.01) | 1.8x10^-3^ | 0.12 | -0.02 (-0.05, 0.01) | 1.6x10^-3^ | 0.19 |  |  |  |
|  | 5x10^-1^ | -0.02 (-0.05, 0.01) | 1.5x10^-3^ | 0.20 | -0.02 (-0.06, 0.01) | 1.6x10^-3^ | 0.16 |  |  |  |
| Vocabulary | 5x10^-8^ | -0.003 (-0.03, 0.03) | 7.4x10^-4^ | 0.85 | 0.01 (-0.03,0.04) | 7.7x10^-4^ | 0.73 | -0.004 (-0.03, 0.03) | 6.9x10^-4^ |  |
|  | 1x10^-3^ | 0.001 (-0.03, 0.03) | 7.3x10^-4^ | 0.94 | 0.02 (-0.01, 0.05) | 1.2x10^-3^ | 0.19 |  |  |  |
|  | 5x10^-2^ | 0.01 (-0.02, 0.04) | 7.8x10^-4^ | 0.66 | 0.005 (-0.03, 0.04) | 7.6x10^-4^ | 0.76 |  |  | 0.79 |
|  | 1x10^-1^ | -0.005 (-0.04, 0.03) | 7.6x10^-4^ | 0.76 | -0.02 (-0.05, 0.01) | 1.1x10^-3^ | 0.26 |  |  |  |
|  | 5x10^-1^ | 0.001 (-0.02, 0.04) | 7.8x10^-4^ | 0.66 | -0.02 (-0.05, 0.02) | 9.5x10^-4^ | 0.35 |  |  |  |
| Matrix Reasoning | 5x10^-8^ | -0.01 (-0.04, 0.02) | 7.7x10^-4^ | 0.57 | 0.01 (-0.03, 0.04) | 7.3x10^-4^ | 0.71 | -0.01 (-0.04, 0.02) | 8.1x10^-4^ |  |
|  | 1x10^-3^ | -0.01 (-0.04, 0.02) | 7.8x10^-4^ | 0.55 | 0.01 (-0.02, 0.04) | 7.4x10^-4^ | 0.66 |  |  |  |
|  | 5x10^-2^ | -0.02 (-0.05, 0.01) | 1.0x10^-3^ | 0.25 | 0.003 (-0.03, 0.03) | 7.0x10^-4^ | 0.87 |  |  | 0.48 |
|  | 1x10^-1^ | -0.03 (-0.06, 0.01) | 1.4x10^-3^ | 0.11 | -0.01 (-0.04, 0.02) | 8.0x10^-4^ | 0.50 |  |  |  |
|  | 5x10^-1^ | -0.04 (-0.07, -0.01) | 2.6x10^-3^ | 0.01 | -0.03 (-0.06, 0.005) | 1.4x10^-3^ | 0.10 |  |  |  |
| CI, confidence interval; IQ, intelligence quotient; PRS, polygenic risk score  Model 1 is based on the PRS including all independent single nucleotide polymorphisms.  Model 2 is based on the PRS including independent single nucleotide polymorphisms, except for the Apolipoprotein (ApoE) region (Chr. 19; 44,400–46,500 kb).  Model 3 is based on the PRS of the two SNPs tagging the Apolipoprotein (ApoE) region (rs7412 and rs429358).  Adjusted p-value threshold=0.0125. | | | | | | | | | |  |

| **Table 9** Association of PRS for Alzheimer’s disease with SDQ results at age 9 | | | | | | | | | | |
| --- | --- | --- | --- | --- | --- | --- | --- | --- | --- | --- |
|  | | **Model 1** | |  | **Model 2** | |  | **Model 3** | |  |
| **Outcome** | **Threshold** | **OR (95% CI)** | **Pseudo-R^2^** | **p** | **OR (95% CI)** | **Pseudo-R^2^** | **p** | **OR (95% CI)** | **Pseudo-R^2^** | **p** |
| Total difficulties | 5x10^-8^ | 1.00 (0.89, 1.14) | 5.3x10^-4^ | 0.95 | 1.01 (0.89, 1.15) | 5.3x10^-4^ | 0.89 | 1.01 (0.89, 1.15)  0.95 (0.84, 1.08)  0.96 (0.84, 1.09)  0.99 (0.87, 1.12)  1.04 (0.91, 1.18) | 5.3x10^-4^  8.3x10^-4^  7.2x10^-4^  5.4 x10^-4^  6.8x10^-4^ | 0.89 |
|  | 1x10^-3^ | 0.99 (0.87, 1.13) | 5.3x10^-4^ | 0.91 | 0.95 (0.84, 1.08) | 8.3x10^-4^ | 0.43 |  |  | 0.43 |
|  | 5x10^-2^ | 1.02 (0.90, 1.16) | 5.6x10^-4^ | 0.78 | 0.96 (0.84, 1.09) | 7.2x10^-4^ | 0.53 |  |  | 0.53 |
|  | 1x10^-1^ | 1.05 (0.92, 1.19) | 8.0x10^-4^ | 0.46 | 0.99 (0.87, 1.12) | 5.4 x10^-4^ | 0.87 |  |  | 0.87 |
|  | 5x10^-1^ | 1.05 (0.92, 1.19) | 7.9x10^-4^ | 0.46 | 1.04 (0.91, 1.18) | 6.8x10^-4^ | 0.57 |  |  | 0.57 |
| Prosocial score | 5x10^-8^ | 0.97 (0.90, 1.04) | 1.4x10^-3^ | 0.36 | 0.91 (0.85, 0.98) | 2.5x10^-3^ | 0.02 | 0.91 (0.85, 0.98)  0.96 (0.89, 1.03)  0.98 (0.91, 1.05)  0.97 (0.90, 1.05)  1.02 (0.95, 1.10) | 2.5x10^-3^  1.5x10^-3^  1.3x10-^2^  1.4x10^-3^  1.3x10^-3^ | 0.02  0.28  0.57  0.43  0.53 |
|  | 1x10^-3^ | 0.98 (0.91, 1.05) | 1.3x10^-3^ | 0.58 | 0.96 (0.89, 1.03) | 1.5x10^-3^ | 0.28 |  |  |  |
|  | 5x10^-2^ | 0.98 (0.91, 1.06) | 1.3x10^-3^ | 0.58 | 0.98 (0.91, 1.05) | 1.3x10-^2^ | 0.57 |  |  |  |
|  | 1x10^-1^ | 0.98 (0.91, 1.05) | 1.3x10^-3^ | 0.53 | 0.97 (0.90, 1.05) | 1.4x10^-3^ | 0.43 |  |  |  |
|  | 5x10^-1^ | 1.00 (0.93, 1.07) | 1.2x10^-3^ | 0.95 | 1.02 (0.95, 1.10) | 1.3x10^-3^ | 0.53 |  |  |  |
| Hyperactivity score | 5x10^-8^ | 0.99 (0.90. 1.09) | 1.3x10^-3^ | 0.86 | 0.96 (0.87, 1.06) | 1.5x10^-3^ | 0.39 | 0.96 (0.87, 1.06)  0.98 (0.89, 1.08)  1.01 (0.92, 1.12)  1.04 (0.94, 1.14)  1.05 (0.95, 1.16) | 1.5x10^-3^  1.3x10^-3^  1.3x10^-3^  1.4x10^-3^  1.6x10^-3^ | 0.39  0.71  0.83  0.47  0.31 |
|  | 1x10^-3^ | 0.99 (0.90, 1.09) | 1.3x10^-3^ | 0.85 | 0.98 (0.89, 1.08) | 1.3x10^-3^ | 0.71 |  |  |  |
|  | 5x10^-2^ | 1.02 (0.92, 1.12) | 1.3x10^-3^ | 0.73 | 1.01 (0.92, 1.12) | 1.3x10^-3^ | 0.83 |  |  |  |
|  | 1x10^-1^ | 1.07 (0.97, 1.18) | 1.8x10^-3^ | 0.18 | 1.04 (0.94, 1.14) | 1.4x10^-3^ | 0.47 |  |  |  |
|  | 5x10^-1^ | 1.05 (0.96. 1.16) | 1.6x10^-3^ | 0.29 | 1.05 (0.95, 1.16) | 1.6x10^-3^ | 0.31 |  |  |  |
| Emotional score | 5x10^-8^ | 0.94 (0.84, 1.05) | 2.9x10^-3^ | 0.25 | 0.99 (0.89, 1.10) | 2.4x10^-3^ | 0.85 | 0.99 (0.89, 1.10)  0.99 (0.89, 1.10)  1.03 (0.93, 1.15)  1.05 (0.95, 1.17)  1.06 (0.95, 1.18) | 2.4x10^-3^  2.3x10^-3^  2.5x10^-3^  2.7x10^-3^  2.8x10^-3^ | 0.85  0.87  0.55  0.35  0.28 |
|  | 1x10^-3^ | 0.94 (0.85, 1.05) | 2.8x10^-3^ | 0.29 | 0.99 (0.89, 1.10) | 2.3x10^-3^ | 0.87 |  |  |  |
|  | 5x10^-2^ | 1.00 (0.90, 1.11) | 2.3x10^-3^ | 0.96 | 1.03 (0.93, 1.15) | 2.5x10^-3^ | 0.55 |  |  |  |
|  | 1x10^-1^ | 1.03 (0.93, 1.15) | 2.5x10^-3^ | 0.58 | 1.05 (0.95, 1.17) | 2.7x10^-3^ | 0.35 |  |  |  |
|  | 5x10^-1^ | 1.07 (0.96, 1.19) | 2.9x10^-3^ | 0.23 | 1.06 (0.95, 1.18) | 2.8x10^-3^ | 0.28 |  |  |  |
| Conduct problems | 5x10^-8^ | 0.96 (0.86, 1.07) | 9.5x10^-4^ | 0.43 | 1.00 (0.90, 1.11) | 7.2x10^-4^ | 0.95 | 1.00 (0.90, 1.11)  0.95 (0.85, 1.05)  0.96 (0.86, 1.06)  0.98 (0.88, 1.09)  1.03 (0.93, 1.14) | 7.2x10^-4^  1.1x10^-3^  9.5x10^-4^  7.7x10^-4^  8.2x10^-4^ | 0.95  0.31  0.43  0.71  0.61 |
|  | 1x10^-3^ | 0.96 (0.87, 1.07) | 9.2x10^-4^ | 0.47 | 0.95 (0.85, 1.05) | 1.1x10^-3^ | 0.31 |  |  |  |
|  | 5x10^-2^ | 0.99 (0.89, 1.10) | 7.3x10^-4^ | 0.88 | 0.96 (0.86, 1.06) | 9.5x10^-4^ | 0.43 |  |  |  |
|  | 1x10^-1^ | 1.03 (0.93, 1.14) | 8.2x10^-4^ | 0.59 | 0.98 (0.88, 1.09) | 7.7x10^-4^ | 0.71 |  |  |  |
|  | 5x10^-1^ | 1.01 (0.91, 1.13) | 7.5x10^-4^ | 0.79 | 1.03 (0.93, 1.14) | 8.2x10^-4^ | 0.61 |  |  |  |
| Peer problems | 5x10^-8^ | 1.01 (0.91, 1.11) | 4.5x10^-4^ | 0.91 | 1.02 (0.93, 1.13) | 5.2x10^-4^ | 0.64 | 1.02 (0.93, 1.13)  0.92 (0.84, 1.01)  0.96 (0.87, 1.06)  0.98 (0.89, 1.08)  1.00 (0.91, 1.10) | 5.2x10^-4^  1.3x10^-3^  6.3x10^-4^  4.9x10^-4^  4.5x10^-4^ | 0.64  0.10  0.45  0.71  1.00 |
|  | 1x10^-3^ | 0.95 (0.87, 1.05) | 7.4x10^-4^ | 0.34 | 0.92 (0.84, 1.01) | 1.3x10^-3^ | 0.10 |  |  |  |
|  | 5x10^-2^ | 0.93 (0.85, 1.03) | 1.1x10^-3^ | 0.16 | 0.96 (0.87, 1.06) | 6.3x10^-4^ | 0.45 |  |  |  |
|  | 1x10^-1^ | 0.94 (0.85, 1.03) | 1.0x10^-3^ | 0.19 | 0.98 (0.89, 1.08) | 4.9x10^-4^ | 0.71 |  |  |  |
|  | 5x10^-1^ | 0.97 (0.88, 1.07) | 5.8x10^-4^ | 0.53 | 1.00 (0.91, 1.10) | 4.5x10^-4^ | 1.00 |  |  |  |
| CI, confidence interval; OR, odds ratio; PRS, polygenic risk score; SDQ, strengths and difficulties questionnaire  Model 1 is based on the PRS including all independent single nucleotide polymorphisms.  Model 2 is based on the PRS including independent single nucleotide polymorphisms, except for the Apolipoprotein (ApoE) region (Chr. 19; 44,400–46,500 kb).  Model 3 is based on the PRS of the two SNPs tagging the Apolipoprotein (ApoE) region (rs7412 and rs429358).  Adjusted p-value threshold=0.0125. | | | | | | | | | | |

| **Table 10** Association of PRS for Alzheimer’s disease with SDQ results at age 12 | | | | | | | | | | |
| --- | --- | --- | --- | --- | --- | --- | --- | --- | --- | --- |
|  |  | **Model 1** | |  | **Model 2** | |  | **Model 3** | |  |
| **Outcome** | **Threshold** | **OR (95% CI)** | **Pseudo-R^2^** | **p** | **OR (95% CI)** | **Pseudo-R^2^** | **p** | **OR (95% CI)** | **Pseudo-R^2^** | **p** |
| Total difficulties | 5x10^-8^ | 1.01 (0.90, 1.14) | 1.7x10^-3^ | 0.85 | 1.03 (0.91, 1.15) | 1.8x10^-3^ | 0.67 | 1.00 (0.89, 1.13) | 1.7x10^-3^ |  |
|  | 1x10^-3^ | 0.98 (0.87, 1.10) | 1.8x10^-3^ | 0.68 | 0.98 (0.88, 1.11) | 1.7x10^-3^ | 0.80 |  |  |  |
|  | 5x10^-2^ | 1.05 (0.93, 1.17) | 2.0 x10^-3^ | 0.46 | 1.02 (0.91, 1.15) | 1.8x10^-3^ | 0.74 |  |  | 0.96 |
|  | 1x10^-1^ | 1.07 (0.95, 1.20) | 2.3 x10^-3^ | 0.24 | 1.04 (0.92, 1.17) | 1.9x10^-3^ | 0.55 |  |  |  |
|  | 5x10^-1^ | 1.04 (0.92, 1.16) | 1.9x10^-3^ | 0.56 | 0.98 (0.87, 1.11) | 1.7x10^-3^ | 0.77 |  |  |  |
| Prosocial score | 5x10^-8^ | 0.93 (0.86, 1.00) | 2.0x10^-3^ | 0.05 | 0.90 (0.83, 0.97) | 3.0x10^-3^ | 0.005 | 0.97 (0.90, 1.05) | 1.3x10^-3^ |  |
|  | 1x10^-3^ | 0.96 (0.89, 1.04) | 1.4x10^-3^ | 0.31 | 1.01 (0.94, 1.09) | 1.2x10^-3^ | 0.77 |  |  |  |
|  | 5x10^-2^ | 1.04 (0.97, 1.13) | 1.4x10^-3^ | 0.28 | 1.03 (0.95, 1.11) | 1.3x10^-3^ | 0.52 |  |  | 0.47 |
|  | 1x10^-1^ | 1.06 (0.99,1.15) | 1.7x10^-3^ | 0.11 | 1.03 (0.96, 1.12) | 1.3x10^-3^ | 0.39 |  |  |  |
|  | 5x10^-1^ | 1.10 (1.02, 1.18) | 2.4x10^-3^ | 0.02 | 1.06 (0.98, 1.14) | 1.7x10^-3^ | 0.14 |  |  |  |
| Hyperactivity score | 5x10^-8^ | 0.97 (0.87, 1.08) | 4.9x10^-4^ | 0.56 | 0.97 (0.87, 1.09) | 4.4x10^-4^ | 0.63 | 0.99 (0.89,1.10) | 3.6x10^-4^ |  |
|  | 1x10^-3^ | 1.01 (0.90, 1.12) | 3.6x10^-4^ | 0.91 | 1.03 (0.92, 1.15) | 4.5x10^-4^ | 0.62 |  |  |  |
|  | 5x10^-2^ | 1.06 (0.95,1.18) | 7.7x10^-4^ | 0.30 | 1.01 (0.90, 1.13) | 3.6x10^-4^ | 0.86 |  |  | 0.86 |
|  | 1x10^-1^ | 1.09 (0.98,1.21) | 1.3x10^-3^ | 0.13 | 1.06 (0.95, 1.18) | 7.6x10^-4^ | 0.31 |  |  |  |
|  | 5x10^-1^ | 1.07 (0.96, 1.20) | 1.0x10^-3^ | 0.20 | 1.05 (0.94, 1.17) | 6.4x10^-4^ | 0.39 |  |  |  |
| Emotional score | 5x10^-8^ | 1.07 (0.96, 1.20) | 1.4x10^-3^ | 0.19 | 1.09 (0.98, 1.22) | 1.7x10^-3^ | 0.12 | 1.05 (0.94,1.17) | 9.4x10^-4^ |  |
|  | 1x10^-3^ | 1.08 (0.97, 1.21) | 1.5x10^-3^ | 0.16 | 1.08 (0.97, 1.21) | 1.4x10^-3^ | 0.17 |  |  |  |
|  | 5x10^-2^ | 1.12 (1.00, 1.25) | 2.3x10^-3^ | 0.05 | 1.08 (0.97, 1.21) | 1.4x10^-3^ | 0.17 |  |  | 0.42 |
|  | 1x10^-1^ | 1.14 (1.02, 1.28) | 2.9x10^-3^ | 0.02 | 1.09 (0.97, 1.22) | 1.6x10^-3^ | 0.13 |  |  |  |
|  | 5x10^-1^ | 1.16 (1.04, 1.26) | 3.5x10^-3^ | 0.01 | 1.06 (0.95, 1.19) | 1.2x10^-3^ | 0.27 |  |  |  |
| Conduct problems | 5x10^-8^ | 0.99 (0.89, 1.10) | 1.6x10^-3^ | 0.81 | 0.99 (0.88,1.10) | 1.6x10^-3^ | 0.78 | 0.99 (0.89,1.10) | 1.6x10^-3^ |  |
|  | 1x10^-3^ | 1.04 (0.93, 1.15) | 1.7x10^-3^ | 0.52 | 1.06 (0.95, 1.18) | 2.0x10^-3^ | 0.31 |  |  |  |
|  | 5x10^-2^ | 1.04 (0.94,1.16) | 1.8x10^-3^ | 0.46 | 1.02 (0.91, 1.13) | 1.6x10^-3^ | 0.74 |  |  | 0.88 |
|  | 1x10^-1^ | 1.06 (0.95,1.18) | 2.0x10^-3^ | 0.31 | 1.03 (0.93, 1.15) | 1.7x10^-3^ | 0.58 |  |  |  |
|  | 5x10^-1^ | 1.05 (0.94, 1.16) | 1.8x10^-3^ | 0.42 | 0.99 (0.89, 1.10) | 1.6x10^-3^ | 0.84 |  |  |  |
| Peer problems | 5x10^-8^ | 0.96 (0.87, 1.06) | 3.0x10^-4^ | 0.43 | 1.06 (0.96, 1.17) | 5.7x10^-4^ | 0.23 | 0.93 (0.84, 1.03) | 7.8x10^-4^ | 0.15 |
|  | 1x10^-3^ | 0.98 (0.89, 1.08) | 1.7x10^-4^ | 0.63 | 0.98 (0.89, 1.08) | 1.6x10^-4^ | 0.64 |  |  |  |
|  | 5x10^-2^ | 0.96 (0.87,1.06) | 3.3x10^-4^ | 0.40 | 0.96 (0.87, 1.06) | 3.1x10^-4^ | 0.42 |  |  |  |
|  | 1x10^-1^ | 0.98 (0.89, 1.08) | 1.7x10^-4^ | 0.63 | 0.95 (0.87, 1.05) | 3.8x10^-4^ | 0.35 |  |  |  |
|  | 5x10^-1^ | 1.00 (0.91, 1.11) | 9.4x10^-5^ | 0.92 | 0.95 (0.86, 1.05) | 4.3x10^-3^ | 0.31 |  |  |  |
| CI, confidence interval; OR, odds ratio; PRS, polygenic risk score; SDQ, strengths and difficulties questionnaire.  Model 1 is based on the PRS including all independent single nucleotide polymorphisms.  Model 2 is based on the PRS including independent single nucleotide polymorphisms, except for the Apolipoprotein (ApoE) region (Chr. 19; 44,400–46,500 kb).  Model 3 is based on the PRS of the two SNPs tagging the Apolipoprotein (ApoE) region (rs7412 and rs429358).  Adjusted p-value threshold=0.0125. | | | | | | | | | | |

| **Table 11** Association of PRS (excluding SNPs associated with educational attainment at 5x10^-8^) with total points for Key Stages 3-5 | | | | | | | |  |
| --- | --- | --- | --- | --- | --- | --- | --- | --- |
|  |  | **Model 1** | |  | **Model 2** | |  |  |
| **Outcome** | **Threshold** | **β-coefficient (95% CI)** | **R^2^** | **p** | **β-coefficient (95% CI)** | **R^2^** | **p** |  |
| Key Stage 3 | 5x10^-8^ | 0.01 (-0.01, 0.04) | 2.2x10^-3^ | 0.33 | 0.01 (-0.02, 0.03) | 2.1x10^-3^ | 0.61 |  |
|  | 1x10^-3^ | 0.01 (-0.02,0.03) | 2.2x10^-3^ | 0.50 | 0.0004 (-0.02, 0.03) | 2.1x10^-3^ | 0.98 |  |
|  | 5x10^-2^ | -0.001 (-0.03, 0.02) | 2.1x10^-3^ | 0.95 | -0.004 (-0.03, 0.02) | 2.1x10^-3^ | 0.77 |  |
|  | 1x10^-1^ | -0.01 (-0.04, 0.01) | 2.2x10^-3^ | 0.37 | -0.01 (-0.04, 0.01) | 2.3x10^-3^ | 0.27 |  |
|  | 5x10^-1^ | -0.03 (-0.05, -0.002) | 2.8x10^-3^ | 0.03 | -0.03 (-0.05, -0.004) | 2.9x10^-3^ | 0.02 |  |
| Key Stage 4 | 5x10^-8^ | 0.001 (-0.02, 0.02) | 3.6x10^-3^ | 0.96 | 0.001 (-0.02, 0.02) | 3.6x10^-3^ | 0.95 |  |
|  | 1x10^-3^ | 0.01 (-0.01, 0.03) | 3.6x10^-3^ | 0.85 | 0.01 (-0.01, 0.04) | 3.7x10^-3^ | 0.30 |  |
|  | 5x10^-2^ | -0.01 (-0.03, 0.01) | 3.7x10^-3^ | 0.46 | -0.01 (-0.03, 0.01) | 3.7x10^-3^ | 0.37 |  |
|  | 1x10^-1^ | -0.02 (-0.04, 0.005) | 3.9x10^-3^ | 0.37 | -0.02 (-0.04, 0,004) | 4.0x10^-3^ | 0.11 |  |
|  | 5x10^-1^ | -0.03 (-0.05, -0.002) | 4.2x10^-3^ | 0.11 | -0.03 (-0.05, -0.002) | 4.2x10^-3^ | 0.04 |  |
| Key Stage 5 | 5x10^-8^ | 0.01 (-0.02, 0.04) | 1.6x10^-3^ | 0.55 | -0.0001 (-0.03, 0.03) | 1.5x10^-3^ | 0.99 |  |
|  | 1x10^-3^ | 0.01 (-0.02, 0.04) | 1.6x10^-3^ | 0.56 | 0.004 (-0.03, 0.03) | 1.5x10^-3^ | 0.81 |  |
|  | 5x10^-2^ | -0.01 (-0.04, 0.02) | 1.6x10^-3^ | 0.49 | -0.01 (-0.04, 0.02) | 1.7x10^-3^ | 0.43 |  |
|  | 1x10^-1^ | -0.02 (-0.05, 0.01) | 2.1x10^-3^ | 0.11 | -0.03 (-0.06, 0.004) | 2.2x10^-3^ | 0.09 |  |
|  | 5x10^-1^ | -0.03 (-0.06, 0.004) | 2.2x10^-3^ | 0.09 | -0.03 (-0.06, 0.003) | 2.2x10^-3^ | 0.08 |  |
| CI, confidence interval; PRS, polygenic risk score.  Model 1 is based on the PRS including all independent single nucleotide polymorphisms.  Model 2 is based on the PRS including independent single nucleotide polymorphisms, except for the Apolipoprotein (ApoE) region (Chr. 19; 44,400–46,500 kb).  Adjusted p-value threshold=0.0125.  *.* | | | | | | | | |

| **Table 12** Association of PRS (excluding SNPs associated with educational attainment at 5x10^-2^) with total points for Key Stages 3-5 | | | | | | | |  |
| --- | --- | --- | --- | --- | --- | --- | --- | --- |
|  |  | **Model 1** | |  | **Model 2** | |  |  |
| **Outcome** | **Threshold** | **β-coefficient (95% CI)** | **R^2^** | **p** | **β-coefficient (95% CI)** | **R^2^** | **p** |  |
| Key Stage 3 | 5x10^-8^ | 0.01 (-0.01, 0.04) | 2.2x10^-3^ | 0.34 | 0.003 (-0.02, 0.03) | 2.1x10^-3^ | 0.79 |  |
|  | 1x10^-3^ | 0.01 (-0.02,0.03) | 2.2x10^-3^ | 0.49 | 0.002 (-0.02,0.03) | 2.1x10^-3^ | 0.86 |  |
|  | 5x10^-2^ | 0.01 (-0.01,0.04) | 2.2x10^-3^ | 0.41 | 0.01 (-0.02,0.03) | 2.2x10^-3^ | 0.51 |  |
|  | 1x10^-1^ | -0.0004 (-0.03,0.02) | 2.1x10^-3^ | 0.98 | -0.002 (-0.03, 0.02) | 2.1x10^-3^ | 0.85 |  |
|  | 5x10^-1^ | -0.02 (-0.04,0.01) | 2.4x10^-3^ | 0.18 | -0.02 (-0.04,0.01) | 2.4x10^-3^ | 0.14 |  |
| Key Stage 4 | 5x10^-8^ | 0.002 (-0.02, 0.03) | 3.6x10^-3^ | 0.85 | 0.001 (-0.02, 0.03) | 3.6x10^-3^ | 0.91 |  |
|  | 1x10^-3^ | 0.01 (-0.01,0.04) | 3.7x10^-3^ | 0.36 | 0.02 (-0.01,0.04) | 3.8x10^-3^ | 0.21 |  |
|  | 5x10^-2^ | -0.002 (-0.03,0.02) | 3.6x10^-3^ | 0.85 | -0.002 (-0.03,0.02) | 3.6x10^-3^ | 0.88 |  |
|  | 1x10^-1^ | -0.01 (-0.03,0.01) | 3.7x10^-3^ | 0.39 | -0.01 (-0.03,0.01) | 3.7x10^-3^ | 0.40 |  |
|  | 5x10^-1^ | -0.02 (-0.04,0.01) | 3.9x10^-3^ | 0.15 | -0.02 (-0.04,0.01) | 3.9x10^-3^ | 0.16 |  |
| Key Stage 5 | 5x10^-8^ | 0.01 (-0.02, 0.04) | 1.7x10^-3^ | 0.39 | 0.0003 (-0.03, 0.03) | 1.5x10^-3^ | 0.98 |  |
|  | 1x10^-3^ | 0.01 (-0.02,0.04) | 1.6x10^-3^ | 0.49 | 0.01 (-0.02, 0.04) | 1.5x10^-3^ | 0.72 |  |
|  | 5x10^-2^ | -0.01 (-0.04,0.02) | 1.6x10^-3^ | 0.68 | -0.01 (-0.04, 0.02) | 1.6x10^-3^ | 0.61 |  |
|  | 1x10^-1^ | -0.02 (-0.05, 0.01) | 1.9x10^-3^ | 0.22 | -0.02 (-0.05, 0.01) | 1.9x10^-3^ | 0.18 |  |
|  | 5x10^-1^ | -0.03 (-0.06,0.005) | 2.2x10^-3^ | 0.10 | -0.03 (-0.06, 0.004) | 2.2x10^-3^ | 0.09 |  |
| CI, confidence interval; PRS, polygenic risk score.  Model 1 is based on the PRS including all independent single nucleotide polymorphisms.  Model 2 is based on the PRS including independent single nucleotide polymorphisms, except for the Apolipoprotein (ApoE) region (Chr. 19; 44,400–46,500 kb).  Adjusted p-value threshold=0.0125.  *.* | | | | | | | | |

| **Table 13** Association of PRS for Alzheimer’s disease (excluding SNPs associated with educational attainment at 5x10^-8^) and odds of sitting Key Stage 5 exams | | | | | |  |
| --- | --- | --- | --- | --- | --- | --- |
|  | **Model 1** | | | **Model 2** | | |
| **Threshold** | **OR (95% CI)** | **Pseudo-R^2^** | **p** | **OR (95% CI)** | **Pseudo-R^2^** | **p** |
| 5x10^-8^ | 0.95 (0.85, 1.05) | 1.0x10^-3^ | 0.30 | 1.02 (0.92, 1.14) | 6.7x10^-4^ | 0.67 |
| 1x10^-3^ | 0.97 (0.87, 1.07) | 7.7x10^-4^ | 0.51 | 1.00 (0.90,1.11) | 6.0x10^-4^ | 0.96 |
| 5x10^-2^ | 0.98 (0.88, 1.09) | 6.4x10^-4^ | 0.75 | 0.99 (0.89, 1.10) | 6.1x10^-4^ | 0.88 |
| 1x10^-1^ | 0.96 (0.86, 1.07) | 8.3x10^-4^ | 0.44 | 0.97 (0.87,1.07)) | 7.7x10^-4^ | 0.52 |
| 5x10^-1^ | 0.94 (0.85, 1.05) | 1.1x10^-3^ | 0.26 | 0.94 (0.85, 1.05) | 1.0x10^-3^ | 0.29 |
| CI, confidence interval; OR, odds ratio; PRS, polygenic risk score.  Model 1 is based on the PRS including all independent single nucleotide polymorphisms.  Model 2 is based on the PRS including independent single nucleotide polymorphisms, except for the Apolipoprotein (ApoE) region (Chr. 19; 44,400–46,500 kb).  Adjusted p-value threshold=0.0125. | | | | | | |

| **Table 14** Association of PRS for Alzheimer’s disease (excluding SNPs associated with educational attainment at 5x10^-2^) and odds of sitting Key Stage 5 exams | | | | | |  |
| --- | --- | --- | --- | --- | --- | --- |
|  | **Model 1** | | | **Model 2** | | |
| **Threshold** | **OR (95% CI)** | **Pseudo-R^2^** | **p** | **OR (95% CI)** | **Pseudo-R^2^** | **p** |
| 5x10^-8^ | 0.96 (0.86, 1.07) | 8.3x10^-4^ | 0.44 | 1.01 (0.90, 1.12) | 6.0x10^-4^ | 0.92 |
| 1x10^-3^ | 0.99 (0.89, 1.11) | 6.1x10^-4^ | 0.91 | 1.02 (0.92,1.13) | 6.4x10^-4^ | 0.95 |
| 5x10^-2^ | 0.99 (0.89, 1.10) | 6.3x10^-4^ | 0.80 | 0.99 (0.89, 1.10) | 6.1x10^-4^ | 0.86 |
| 1x10^-1^ | 0.96 (0.86, 1.06) | 8.5x10^-4^ | 0.42 | 0.98 (0.88, 1.09) | 6.7x10^-4^ | 0.51 |
| 5x10^-1^ | 0.93 (0.84, 1.04) | 1.2x10^-3^ | 0.21 | 0.95 (0.85, 1.05) | 1.0x10^-3^ | 0.29 |
| CI, confidence interval; OR, odds ratio; PRS, polygenic risk score.  Model 1 is based on the PRS including all independent single nucleotide polymorphisms.  Model 2 is based on the PRS including independent single nucleotide polymorphisms, except for the Apolipoprotein (ApoE) region (Chr. 19; 44,400–46,500 kb).  Adjusted p-value threshold=0.0125. | | | | | | |

| **Table 15** Association of PRS (excluding SNPs associated with educational attainment at 5x10^-8^) for Alzheimer’s disease with cognitive measures at age 8 | | | | | | | | |
| --- | --- | --- | --- | --- | --- | --- | --- | --- |
|  |  | **Model 1** | | | **Model 2** | | |  |
| **Outcome** | **Threshold** | **β-coefficient**  **(95% CI)** | **R^2^** | **p** | **β-coefficient**  **(95% CI)** | **R^2^** | **p** |  |
| Total IQ | 5x10^-8^ | -0.001 (-0.03,0.03) | 2.7x10^-4^ | 0.99 | 0.003 (-0.02, 0.03) | 2.8x10^-4^ | 0.82 |  |
|  | 1x10^-3^ | -0.01 (-0.03, 0.02) | 3.4x10^-4^ | 0.54 | -0.01 (-0.04, 0.01) | 4.2x10^-4^ | 0.37 |  |
|  | 5x10^-2^ | -0.03 (-0.06, -0.01) | 1.4x10^-3^ | 0.02 | -0.03 (-0.06, -0.01) | 1.5x10^-3^ | 0.01 |  |
|  | 1x10^-1^ | -0.04 (-0.07, -0.02) | 2.0x10^-3^ | 0.002 | -0.04 (-0.07, -0.02) | 2.1x10^-3^ | 0.002 |  |
|  | 5x10^-1^ | -0.04 (-0.07, -0.01) | 2.0x10^-3^ | 0.002 | -0.04 (-0.07, -0.02) | 2.0x10^-3^ | 0.002 |  |
| Verbal IQ | 5x10^-8^ | 0.01 (-0.02, 0.03) | 6.2x10^-4^ | 0.64 | 0.02 (-0.01, 0.05) | 9.4x10^-4^ | 0.16 |  |
|  | 1x10^-3^ | 0.005 (-0.02, 0.03) | 6.0x10^-4^ | 0.73 | 0.01 (-0.02, 0.03) | 6.1x10^-4^ | 0.66 |  |
|  | 5x10^-2^ | -0.02 (-0.05, 0.003) | 1.2 x10^-3^ | 0.08 | -0.03 (-0.05, 0.001) | 1.2x10^-3^ | 0.06 |  |
|  | 1x10^-1^ | -0.04 (-0.06, -0.01) | 1.8x10^-3^ | 0.01 | -0.04 (-0.06, -0.01) | 1.9x10^-3^ | 0.01 |  |
|  | 5x10^-1^ | -0.04 (-0.07, -0.01) | 2.1x10^-3^ | 0.004 | -0.04 (-0.07, -0.01) | 2.2x10^-3^ | 0.003 |  |
| Performance IQ | 5x10^-8^ | -0.004 (-0.03, 0.02) | 2.4x10^-4^ | 0.74 | -0.01 (-0.04, 0.01) | 4.4x10^-4^ | 0.28 |  |
|  | 1x10^-3^ | -0.02 (-0.04, 0.01) | 5.5x10^-4^ | 0.19 | -0.03 (-0.05, 0.001) | 8.9x10^-4^ | 0.06 |  |
|  | 5x10^-2^ | -0.03 (-0.06, -0.01) | 1.4x10^-3^ | 0.01 | -0.04 (-0.06, -0.01) | 1.5x10^-3^ | 0.01 |  |
|  | 1x10^-1^ | -0.04 (-0.07, -0.01) | 1.8x10^-3^ | 0.004 | -0.04 (-0.07, -0.01) | 1.8x10^-3^ | 0.003 |  |
|  | 5x10^-1^ | -0.03 (-0.06, -0.01) | 1.3x10^-3^ | 0.02 | -0.03 (-0.06, -0.01) | 1.3x10^-3^ | 0.02 |  |

| CI, confidence interval; IQ, intelligence quotient; PRS, polygenic risk score.  Model 1 is based on the PRS including all independent single nucleotide polymorphisms.  Model 2 is based on the PRS including independent single nucleotide polymorphisms, except for the Apolipoprotein (ApoE) region (Chr. 19; 44,400–46,500 kb).  Adjusted p-value threshold=0.0125 |
| --- |

|  |
| --- |

| **Table 16** Association of PRS (excluding SNPs associated with educational attainment at 5x10^-2^) for Alzheimer’s disease with cognitive measures at age 8 | | | | | | | | |
| --- | --- | --- | --- | --- | --- | --- | --- | --- |
|  |  | **Model 1** | | | **Model 2** | | |  |
| **Outcome** | **Threshold** | **β-coefficient**  **(95% CI)** | **R^2^** | **p** | **β-coefficient**  **(95% CI)** | **R^2^** | **p** |  |
| Total IQ | 5x10^-8^ | 0.003 (-0.02,0.03) | 2.8x10^-4^ | 0.81 | -0.001 (-0.03,0.03) | 2.7x10^-4^ | 0.96 |  |
|  | 1x10^-3^ | -0.01 (-0.03, 0.02) | 3.1x10^-4^ | 0.66 | -0.01 (-0.04, 0.02) | 3.1x10^-4^ | 0.48 |  |
|  | 5x10^-2^ | -0.03 (-0.05, -0.001) | 1.0x10^-3^ | 0.04 | -0.03 (-0.06, -0.002) | 1.0x10^-3^ | 0.03 |  |
|  | 1x10^-1^ | -0.04 (-0.06, -0.01) | 1.5x10^-3^ | 0.01 | -0.03 (-0.06, -0.01) | 1.5x10^-3^ | 0.01 |  |
|  | 5x10^-1^ | -0.04 (-0.06, -0.01) | 1.6x10^-3^ | 0.01 | -0.04 (-0.06, -0.01) | 1.6x10^-3^ | 0.01 |  |
| Verbal IQ | 5x10^-8^ | 0.01 (-0.02, 0.03) | 6.5x10^-4^ | 0.52 | -0.01 (-0.01, 0.04) | 7.8x10^-4^ | 0.30 |  |
|  | 1x10^-3^ | 0.01 (-0.02, 0.04) | 7.0x10^-4^ | 0.41 | 0.01 (-0.02, 0.04) | 6.9x10^-4^ | 0.44 |  |
|  | 5x10^-2^ | -0.02 (-0.04, 0.01) | 8.7x10^-4^ | 0.21 | -0.02 (-0.05, 0.01) | 9.3x10^-4^ | 0.17 |  |
|  | 1x10^-1^ | -0.03 (-0.05, -0.001) | 1.4x10^-3^ | 0.04 | -0.03 (-0.06, -0.003) | 1.5x10^-3^ | 0.03 |  |
|  | 5x10^-1^ | -0.03 (-0.06, -0.01) | 1.7x10^-3^ | 0.02 | -0.03 (-0.06, -0.01) | 1.7x10^-3^ | 0.01 |  |
| Performance IQ | 5x10^-8^ | -0.001 (-0.03, 0.03) | 2.2x10^-4^ | 0.92 | -0.02 (-0.04, 0.01) | 4.7x10^-4^ | 0.25 |  |
|  | 1x10^-3^ | -0.02 (-0.05, 0.01) | 6.7x10^-4^ | 0.12 | -0.03 (-0.05, 0.001) | 9.0x10^-4^ | 0.06 |  |
|  | 5x10^-2^ | -0.03 (-0.06, -0.01) | 1.3x10^-3^ | 0.02 | -0.03 (-0.06, -0.01) | 1.3x10^-3^ | 0.01 |  |
|  | 1x10^-1^ | -0.03 (-0.06, -0.01) | 1.4x10^-3^ | 0.01 | -0.04 (-0.06, -0.01) | 1.5x10^-3^ | 0.01 |  |
|  | 5x10^-1^ | -0.03 (-0.06, -0.003) | 1.1x10^-3^ | 0.03 | -0.03 (-0.06, -0.003) | 1.1x10^-3^ | 0.03 |  |
| CI, confidence interval; OR, odds ratio; PRS, polygenic risk score.  Model 1 is based on the PRS including all independent single nucleotide polymorphisms.  Model 2 is based on the PRS including independent single nucleotide polymorphisms, except for the Apolipoprotein (ApoE) region (Chr. 19; 44,400–46,500 kb).  Adjusted p-value threshold=0.0125. | | | | | | | |  |

| **Table 17** Association of PRS (excluding SNPs associated with educational attainment at 5x10^-8^) for Alzheimer’s disease with cognitive measures at age 15 | | | | | | | |  |
| --- | --- | --- | --- | --- | --- | --- | --- | --- |
|  |  | **Model 1** | | | **Model 2** | | | |
| **Outcome** | **Threshold** | **β-coefficient**  **(95% CI)** | **R^2^** | **p** | **β-coefficient**  **(95% CI)** | **R^2^** | **p** | |
| Total IQ | 5x10^-8^ | -0.01 (-0.04, 0.02) | 1.3x10^-3^ | 0.44 | 0.01 (-0.02, 0.05) | 1.1x10^-3^ | 0.68 | |
|  | 1x10^-3^ | -0.01 (-0.04, 0.02) | 1.2x10^-3^ | 0.45 | -0.01 (-0.04, 0.03) | 1.1x10^-3^ | 0.70 | |
|  | 5x10^-2^ | -0.01 (-0.05, 0.02) | 1.3x10^-3^ | 0.44 | -0.01 (-0.04, 0.02) | 1.2x10^-3^ | 0.53 | |
|  | 1x10^-1^ | -0.02 (-0.06, 0.01) | 1.7x10^-3^ | 0.14 | -0.02 (-0.06, 0.01) | 1.6x10^-3^ | 0.17 | |
|  | 5x10^-1^ | -0.02 (-0.05, 0.01) | 1.5x10^-3^ | 0.24 | -0.02 (-0.05, 0.01) | 1.4x10^-3^ | 0.27 | |
| Vocabulary IQ | 5x10^-8^ | -0.003 (-0.03, 0.03) | 7.4x10^-4^ | 0.85 | 0.01 (-0.03,0.04) | 7.7x10^-4^ | 0.73 | |
|  | 1x10^-3^ | 0.002 (-0.03,0.03) | 7.4x10^-4^ | 0.91 | 0.002 (-0.03,0.03) | 7.4x10^-4^ | 0.91 | |
|  | 5x10^-2^ | 0.01 (-0.02, 0.04) | 8.1x10^-4^ | 0.59 | 0.01(-0.02, 0.04) | 8.1x10^-4^ | 0.59 | |
|  | 1x10^-1^ | -0.003 (-0.03, 0.03) | 7.4x10^-4^ | 0.83 | -0.004 (-0.03,0.03) | 7.5x10^-4^ | 0.82 | |
|  | 5x10^-1^ | 0.01 (-0.02, 0.04) | 8.1x10^-4^ | 0.59 | 0.01 (-0.02, 0.04) | 8.0x10^-4^ | 0.60 | |
| Matrix Reasoning | 5x10^-8^ | -0.01 (-0.04, 0.02) | 7.7x10^-4^ | 0.57 | 0.01 (-0.03, 0.04) | 7.3x10^-4^ | 0.71 | |
|  | 1x10^-3^ | -0.01 (-0.04, 0.02) | 7.8x10^-4^ | 0.54 | -0.02 (-0.05, 0.02) | 9.2x10^-4^ | 0.34 | |
|  | 5x10^-2^ | -0.02 (-0.05, 0.01) | 9.8x10^-4^ | 0.29 | -0.02 (-0.05, 0.01) | 9.8x10^-4^ | 0.28 | |
|  | 1x10^-1^ | -0.02 (-0.06, 0.01) | 1.3x10^-3^ | 0.12 | -0.02 (-0.06, 0.01) | 1.3x10^-3^ | 0.12 | |
|  | 5x10^-1^ | -0.04 (-0.07, -0.01) | 2.5x10^-3^ | 0.01 | -0.04 (-0.07, -0.01) | 2.5x10^-3^ | 0.01 | |
| CI, confidence interval; IQ, intelligence quotient; PRS, polygenic risk score.  Model 1 is based on the PRS including all independent single nucleotide polymorphisms.  Model 2 is based on the PRS including independent single nucleotide polymorphisms, except for the Apolipoprotein (ApoE) region (Chr. 19; 44,400–46,500 kb).  Adjusted p-value threshold=0.0125. | | | | | | | | |

| **Table 18** Association of PRS (excluding SNPs associated with educational attainment at 5x10^-2^) for Alzheimer’s disease with cognitive measures at age 15 | | | | | | | |  |
| --- | --- | --- | --- | --- | --- | --- | --- | --- |
|  |  | **Model 1** | | | **Model 2** | | | |
| **Outcome** | **Threshold** | **β-coefficient**  **(95% CI)** | **R^2^** | **p** | **β-coefficient**  **(95% CI)** | **R^2^** | **p** | |
| Total IQ | 5x10^-8^ | -0.01 (-0.04, 0.02) | 1.2x10^-3^ | 0.48 | -0.002 (-0.03, 0.03) | 1.1x10^-3^ | 0.92 | |
|  | 1x10^-3^ | -0.01 (-0.04, 0.02) | 1.2x10^-3^ | 0.64 | 0.0003 (-0.03, 0.03) | 1.1x10^-3^ | 0.98 | |
|  | 5x10^-2^ | -0.01 (-0.04, 0.02) | 1.2x10^-3^ | 0.61 | -0.01 (-0.04, 0.03) | 1.1x10^-3^ | 0.71 | |
|  | 1x10^-1^ | -0.02 (-0.05, 0.01) | 1.5x10^-3^ | 0.19 | -0.02 (-0.05, 0.01) | 1.5x10^-3^ | 0.23 | |
|  | 5x10^-1^ | -0.02 (-0.05, 0.01) | 1.5x10^-3^ | 0.21 | -0.02 (-0.05, 0.01) | 1.5x10^-3^ | 0.23 | |
| Vocabulary IQ | 5x10^-8^ | 0.002 (-0.03, 0.03) | 7.4x10^-4^ | 0.90 | -0.003 (-0.03,0.03) | 7.4x10^-4^ | 0.83 | |
|  | 1x10^-3^ | 0.003 (-0.03,0.03) | 7.4x10^-4^ | 0.86 | 0.01 (-0.03,0.04) | 7.7x10^-4^ | 0.72 | |
|  | 5x10^-2^ | 0.01 (-0.02, 0.04) | 8.2x10^-4^ | 0.56 | 0.01 (-0.02, 0.04) | 8.3x10^-4^ | 0.53 | |
|  | 1x10^-1^ | -0.01 (-0.04, 0.03) | 7.7x10^-4^ | 0.71 | -0.01 (-0.04,0.03) | 7.6x10^-4^ | 0.73 | |
|  | 5x10^-1^ | 0.005 (-0.03, 0.04) | 7.6x10^-4^ | 0.76 | 0.01 (-0.03, 0.04) | 7.6x10^-4^ | 0.75 | |
| Matrix Reasoning | 5x10^-8^ | -0.01 (-0.04, 0.02) | 8.1x10^-4^ | 0.50 | 0.004 (-0.03, 0.03) | 7.0x10^-4^ | 0.83 | |
|  | 1x10^-3^ | -0.01 (-0.04, 0.02) | 7.5x10^-4^ | 0.63 | -0.01 (-0.04, 0.02) | 8.6x10^-4^ | 0.41 | |
|  | 5x10^-2^ | -0.01 (-0.04, 0.02) | 8.5x10^-4^ | 0.43 | -0.01 (-0.04, 0.02) | 8.6x10^-4^ | 0.41 | |
|  | 1x10^-1^ | -0.02 (-0.05, 0.01) | 1.0x10^-3^ | 0.26 | -0.02 (-0.05, 0.01) | 1.0x10^-3^ | 0.25 | |
|  | 5x10^-1^ | -0.04 (-0.07, -0.01) | 2.2x10^-3^ | 0.01 | -0.04 (-0.07, -0.01) | 2.3x10^-3^ | 0.01 | |
| CI, confidence interval; IQ, intelligence quotient; PRS, polygenic risk score.  Model 1 is based on the PRS including all independent single nucleotide polymorphisms.  Model 2 is based on the PRS including independent single nucleotide polymorphisms, except for the Apolipoprotein (ApoE) region (Chr. 19; 44,400–46,500 kb).  Adjusted p-value threshold=0.0125. | | | | | | | | |

**References:**

1. Fraser A, Macdonald-wallis C, Tilling K, Boyd A, Golding J, Davey Smith G, et al. Cohort Profile: the Avon Longitudinal Study of Parents and Children: ALSPAC mothers cohort. *International Journal of Epidemiology*. 2013;**42**(1):97–110.

2. Boyd A, Golding J, Macleod J, Lawlor DA, Fraser A, Henderson J, et al. Cohort Profile: the “children of the 90s”--the index offspring of the Avon Longitudinal Study of Parents and Children. *International Journal of Epidemiology*. 2013 Feb;**42**(1):111–27.

3. Lambert J-C, Ibrahim-Verbaas CA, Harold D, Naj AC, Sims R, Bellenguez C, et al. Meta-analysis of 74,046 individuals identifies 11 new susceptibility loci for Alzheimer’s disease. *Nature Genetics*. 2013;**45**(12):1452–8.

4. Harold D, Abraham R, Hollingworth P, Sims R, Gerrish A, Hamshere ML, et al. Genome-wide association study identifies variants at CLU and PICALM associated with Alzheimer’s disease. *Nature Genetics*. 2009;**41**(10):1088–93.

5. Hollingworth P, Harold D, Sims R, Gerrish A, Lambert J-C, Carrasquillo MM, et al. Common variants at ABCA7, MS4A6A/MS4A4E, EPHA1, CD33 and CD2AP are associated with Alzheimer’s disease. *Nature genetics*. 2011 May;**43**(5):429–35.

6. Seshadri S, Beiser A, Selhub J, Jacques PF, Rosenberg IH, D’Agostino RB, et al. Plasma homocysteine as a risk factor for dementia and Alzheimer’s disease. *The New England journal of medicine*. 2002;**346**(7):476–83.

7. Naj AC, Jun G, Beecham GW, Wang L-S, Vardarajan BN, Buros J, et al. Common variants at MS4A4/MS4A6E, CD2AP, CD33 and EPHA1 are associated with late-onset Alzheimer’s disease. *Nat Genet*. 2011;**43**(5):436–41.

8. Howie BN, Donnelly P, Marchini J. A flexible and accurate genotype imputation method for the next generation of genome-wide association studies. *PLoS Genetics*. 2009;**5**(6).

9. Li Y, Willer CJ, Ding J, Scheet P, Abecasis GR. MaCH: Using sequence and genotype data to estimate haplotypes and unobserved genotypes. *Genetic Epidemiology*. 2010;**34**(8):816–34.

10. Purcell S, Neale B, Todd-Brown K, Thomas L, Ferreira MAR, Bender D, et al. PLINK: A Tool Set for Whole-Genome Association and Population-Based Linkage Analyses. *The American Journal of Human Genetics*. 2007;**81**(3):559–75.

11. Iyegbe C, Campbell D, Butler A, Ajnakina O, Sham P. The emerging molecular architecture of schizophrenia, polygenic risk scores and the clinical implications for gXe research. *Social Psychiatry and Psychiatric Epidemiology*. 2014;**49**(10):1531–4.
